# Qualitative and quantitative analyses of chemical constituents *in vitro* and *in vivo* and systematic evaluation of the pharmacological effects of Tibetan medicine Zhixue Zhentong capsules

**DOI:** 10.3389/fphar.2023.1204947

**Published:** 2023-07-17

**Authors:** Yinglian Song, Yan Liang, Rong Zeng, Ran Li, You Zhou, Sheng Huang, Xiaoli Li, Ning Zhang, Min Xu, Kaipeng Xiong, Ke Fu, Huixuan Ye, Lei Wu, Shaopeng Yu, Wanyue Chen, Ce Tang, Miao Jiang, Zhang Wang

**Affiliations:** ^1^ College of Pharmacy, Chengdu University of Traditional Chinese Medicine, Chengdu, China; ^2^ State Key Laboratory of Southwestern Chinese Medicine Resources, Chengdu University of Traditional Chinese Medicine, Chengdu, China; ^3^ Chengdu Jiuzhitang Jinding Pharmaceutical Company Limited, Chengdu, China; ^4^ College of Ethnomedicine, Chengdu University of Traditional Chinese Medicine, Chengdu, China; ^5^ Jiuzhitang Company Limited, Changsha, China

**Keywords:** *Lamiophlomis rotata* (Benth.) Kudo, UPLC-Q-TOF-MS, metabolites entered in the blood, HPLC, content determination, hemostasis, analgesia, anti-inflammation

## Abstract

**Introduction:** Zhixue Zhentong capsules (ZXZTCs) are a Tibetan medicine preparation solely composed of *Lamiophlomis rotata* (Benth.) Kudo. *L. rotata* is the only species of the genus *Laniophlomis* (family Lamiaceae) that has medicinal constituents derived from the grass or root and rhizome. *L. rotata* is one of the most extensively used folk medicines by Tibetan, Mongolian, Naxi, and other ethnic groups in China and has been listed as a first-class endangered Tibetan medicine. The biological effects of the plant include hemostasis, analgesia, and the removal of blood stasis and swelling.

**Purpose:** This study aimed to profile the overall metabolites of ZXZTCs and those entering the blood. Moreover, the contents of six metabolites were measured and the hemostatic, analgesic, and anti-inflammatory effects of ZXZTCs were explored.

**Methods:** Ultra-performance liquid chromatography–tandem quadrupole time-of-flight high-resolution mass spectrometry (UPLC-Q-TOF-MS) was employed for qualitative analysis of the metabolites of ZXZTCs and those entering the blood. Six metabolites of ZXZTCs were quantitatively determined via high-performance liquid chromatography The hemostatic, analgesic, and anti-inflammatory effects of ZXZTCs were evaluated in various animal models.

**Results:** A total of 36 metabolites of ZXZTCs were identified, including 13 iridoid glycosides, 9 flavonoids, 9 phenylethanol glycosides, 4 phenylpropanoids, and 1 other metabolite. Overall, 11 metabolites of ZXZTCs entered the blood of normal rats. Quantitative analysis of the six main metabolites, shanzhiside methyl ester, chlorogenic acid, 8-O-acetyl shanzhiside methyl ester, forsythin B, luteoloside, and verbascoside, was extensively performed. ZXZTCs exerted hemostatic effects by reducing platelet aggregation and thrombosis and shortening bleeding time. Additionally, ZXZTCs clearly had an analgesic effect, as observed through the prolongation of the latency of writhing, reduction in writhing, and increase in the pain threshold of experimental rats. Furthermore, significant anti-inflammatory effects of ZXZTCs were observed, including a reduction in capillary permeability, the inhibition of foot swelling, and a reduction in the proliferation of granulation tissue.

**Conclusion:** Speculative identification of the overall metabolites of ZXZTCs and those entering the blood can provide a foundation for determining its biologically active constituents. The established method is simple and reproducible and can help improve the quality control level of ZXZTCs as a medicinal product. Evaluating the hemostatic, analgesic, and anti-inflammatory activities of ZXZTCs can help reveal its mechanism.

## Graphical Abstract

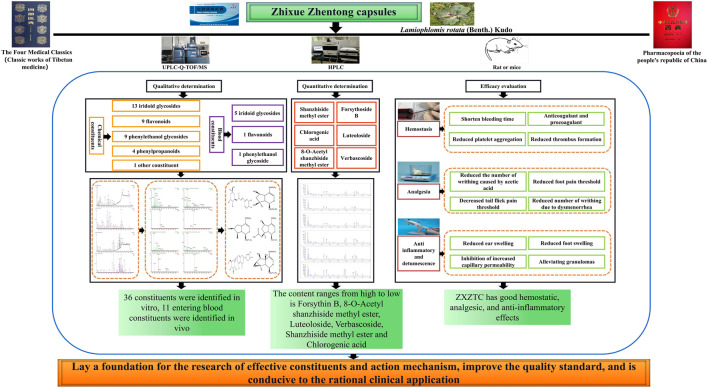

## 1 Introduction

*Lamiophlomis* *rotata* (Benth.) Kudo (Tibetan name: ‘
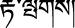
’, transliteration: “Daba, Daub, Dababa”) is the only species of the genus *Laniophlomis* (family Lamiaceae). The grass, root, and rhizome are the main medicinal parts of *Lamiophlomis rotata*. The plant was first published in the famous Tibetan medical works *Si Bu Yi Dian* (early eighth century A.D.) by Yutuo Yuandangongbu and *Yue Wang Yao Zhen* (middle eighth century A.D.) (Zhang et al., 2011). *L. rotata* is a commonly used folk medicine by Tibetan, Mongolian, Naxi, and other ethnic groups in China and is listed as a first-class endangered Tibetan medicine. The effects of *L. rotata* include hemostasis, analgesia, and the removal of blood stasis and swelling (Fu et al., 2019). The plant is currently included in the *Pharmacopoeia of the People’s Republic of China* (National Pharmacopoeia Committee, 2020). Whole grass of *L. rotata* is used to treat *Huangshui* disease, fracture, traumatic injury, osteomyelitis, trauma, and bloating-associated pain. Its roots or rhizomes are used for promoting blood circulation, removing blood stasis, reducing swelling and pain, waist pain, and Qi stagnation (Jia et al., 2016). The sole medicinal material constituent of Zhixue Zhentong capsules (ZXZTCs) is *L. rotata*, which is primarily used to treat bleeding after family planning surgery (installation or removal of the birth control ring and inducing abortion), dysmenorrhea, functional uterine bleeding, traumatic injury, fracture, lumbar sprain, and other types of pain. The main metabolites of *L. rotata* include flavonoids, iridoids, phenylethanol glycosides, and phenylpropanoids. Flavonoids in *L. rotata*, of which luteoloside is the representative metabolite, have anti-inflammatory effects (Mao, 2016). Shanzhiside methyl ester and 8-O-acetyl shanzhiside methyl ester are representative iridoid metabolites that exert analgesic, anti-inflammatory, and hemostatic effects (Zhu et al., 2012). Forsythin B and verbascoside are phenylethanol glycosides that display analgesic activity (Bai et al., 2015). Chlorogenic acid is a phenylpropanoid that acts to reduce the production of inflammatory factors and free radicals to suppress the inflammatory response (Wang et al., 2020; Zhou et al., 2021).

The exertion of a curative effect is dependent on the constituents, highlighting the importance of characterizing the metabolite components of medicinal plants. Luteoloside, shanzhiside methyl ester, 8-O-acetyl shanzhiside methyl ester, forsythin B, verbascoside, and chlorogenic acid are key potential metabolites of choice for determining activity indicators in *L. rotata*.

Wang. et al. (2018) identified 51 metabolites in *L. rotata* via ultra-performance liquid chromatography (UPLC)–quadrupole time-of-flight (Q-TOF)–high-resolution mass spectrometry (MS). Wu et al. (2016) used LC-Q-TOF/MS technology to identify 42 metabolites in *L. rotata*. In similar studies, La et al. (2015) identified 48 metabolites and Zan et al. (2018) uncovered 30 metabolites in *L. rotata* via LC-TOF/MS analysis. Although the metabolites of *L. rotata* have been extensively investigated, no studies have focused on the metabolic profiles of the medicinal preparations of *L. rotata*, such as ZXZTCs.

Based on the results of *in vitro* studies, we analyzed the metabolites of ZXZTCs that enter the blood for the first time in this study, providing a reference for follow-up investigations of the effective constituents and their therapeutic mechanisms of action. LC-MS technology is an effective tool for qualitative analysis. In view of its simple, rapid, and accurate characteristics, UPLC-Q-TOF-MS was used in this study. Using high-resolution UPLC and MS, critical information, such as retention time, can be calculated quickly and accurately, and the molecular mass and fragment ions can be detected and collected precisely (Wu et al., 2016).

The contents of various metabolites (luteoloside, shanzhiside methyl ester, 8-O-acetyl shanzhiside methyl ester, forsythin B, verbascoside, chlorogenic acid, sesamoside, rutin, quercetin, and ergosterol glycoside) of Duyiwei capsules and *Lamiophlomis otate* have been determined via high-performance liquid chromatography (HPLC) in earlier reports (Zhong et al., 2014; Gao et al., 2015; Zhou et al., 2021; Guo et al., 2017). However, to our knowledge, no studies have focused on determining the content of the Tibetan medicinal preparation of *L. rotata*, ZXZTCs. To comprehensively and accurately reflect the quality of ZXZTCs as a medicinal preparation, the contents of six active metabolites were determined via HPLC for the first time, including two iridoid glycosides (shanzhiside methyl ester and 8-O-acetyl shanzhiside methyl ester), two phenylethanol glycosides (forsythin B and verbascoside), one flavone (luteoloside), and one phenylpropanoid (chlorogenic acid). The data obtained should aid in addressing the gaps in existing research, further improve the quality control standard of ZXZTCs, and provide a scientific basis for the optimal utilization of the preparation.

Several research groups, including our own, have conducted pharmacological research on ZXZTCs. Li et al (1992a); Li et al. (1992b) verified the hemostatic effect of ZXZTCs based on a series of experiments on rat, mouse, and rabbit models, demonstrating that ZXZTCs reduce tail bleeding time in mice, increase the number of platelets in rats, and shorten coagulation time. The total effective rate of ZXZTCs in the clinical treatment of patients with thrombocytopenia is 87.8%, which is indicative of a better therapeutic effect (Han et al., 1995). In another study, ZXZTCs combined with remifentanil were used to treat 55 patients with *postpartum* pain after cesarean section. Notably, the levels of serum cortisol, HBV, FIB, LBV, plasma viscosity, serum IL-6, CRP, TNF-α, 5-HT, and PRL were decreased to a significant extent and the total effective rate was higher in the experimental group after treatment compared than in the control group. In recent experiments by Li et al. (2021), the degree of reduction in lower limb motor nerve block and analgesic effect were more obvious in the experimental group, supporting a curative effect of ZXZTCs. Moreover, ZXZTCs were used to effectively treat endometrial hyperplasia, primarily inducing a reduction in endometrial thickness and the alleviation of uterine tissue edema in rats (Fu et al., 2019). ZXZTCs protect the ovary and uterus by increasing the release of ovarian estrogen and improving uterine lesions, promoting significant reductions in the uterine coefficient, transparency, and disorder of myometrial smooth muscle cells and interstitial hyperplasia of ovarian tissue, and marked increases in PROG concentration in serum and VEGF protein expression in uterine tissue (Xiong et al., 2020a). Additionally, ZXZTCs show efficacy in inhibiting the contraction of isolated rat uterine smooth muscle primarily through reducing the average muscle tension (Xiong et al., 2020b). Further recent studies have demonstrated that ZXZTCs regulate functional uterine bleeding, reduce uterine bleeding time, improve the hormone level, and promote the residual excretion of uterine villi and decidual cells. During this process, estradiol and progesterone content is significantly increased (Huang et al., 2020). The present study comprehensively explored the hemostatic, analgesic, anti-inflammatory, and swelling effects of ZXZTCs and revealed a ‘dose-effect’ relationship to confirm and expand its existing and potential pharmacological activities, with the aim of providing guidance for rational clinical applications of this medicinal plant preparation.

## 2 Materials and methods

### 2.1 Drugs, standard substances, and reagents

ZXZTCs (batch numbers 191201, 180902, 200801, 200802, 190101, 201101, 190201, and 170301; Guoyao Zhunzi: Z20049006) were purchased from Chengdu Jiuzhitang Jinding Pharmaceutical Co., Ltd., Carbazochrome (batch number 1703002, Guoyao Zhunzi: H32023286) was acquired from Jiangsu Yang Epson Pharmaceutical Co., Ltd., Yunnan Baiyao capsules (batch number ZCA1706, Guoyao Zhunzi: Z53020799) were obtained from Yunnan Baiyao Group Co., Ltd., Prednisone acetate tablets (batch number 171210, Guoyao Zhunzi: H33021207) were purchased from Zhejiang Xianju Pharmaceutical Co., Ltd., and aspirin enteric-coated tablets (batch number BJ40782, Guoyao Zhunzi: J20171021) were obtained from Bayer S.p.A. Rotundine tablets (batch number 180905, Guoyao Zhunzi: H51021203) were purchased from Sichuan Difit Pharmaceutical Co., Ltd., Ibuprofen capsules (batch number 190201, Guoyao Zhunzi: H22026320) were acquired from Changchun Dirui Pharmaceutical Co., Ltd., Chlorphenamine maleate tablets (batch number 20181203, Guoyao Zhunzi: H42020608) were purchased from Huazhong Pharmaceutical Co., Ltd., Sodium chloride injection (batch number M16101406, Guoyao Zhunzi: H51021158) was procured from Sichuan Kelun Pharmaceutical Co., Ltd.

Loganin (batch number PS000656), luteoloside (batch number PS011538), chlorogenic acid (batch number PS000627), verbascoside (batch number PS000442), 8-O-acetyl shanzhiside methyl ester (batch number PS000191), shanzhiside methyl ester (batch number PS000817), and forsythin B (batch number PS010056) were purchased from Chengdu Push Biotechnology Co., Ltd., with an HPLC purity of >97%.

Methanol (analytical grade) was supplied by Sigma-Aldrich Trading Co., Ltd., Acetonitrile (chromatographic grade) was obtained from Tedia Company, Inc (USA) and methanol (chromatographic grade) was procured from Thermo Fisher Scientific Co., Ltd (China). Ultrapure water was used for all experiments.

### 2.2 Instruments and equipment

The following instruments and equipment were used: ultra-performance liquid chromatography column (ACQUITY, American Waters Company), high-resolution mass spectrometer (Q-TOF, American Waters Company), high-performance liquid chromatography column (Agilent 1260, American Agilent company), vertical ultra-low temperature refrigerator (ULT2586-10-V48, American Thermo Company), ultrasonic cleaner (CQ-250, Shanghai Binengxin Co., Ltd.), vortex mixer (XW-80A, Shanghai Chitang Industrial Co., Ltd.), nitrogen-blowing instrument (KL-512, Beijing Kanglin Technology Co., Ltd.), centrifuge (TDZ5-WS, Changsha Xiangyi Centrifuge Instrument Co., Ltd.), platelet aggregation instrument (SC-2000, Beijing Succeeder Technology Development Co., Ltd.), automatic thrombus detector (LMK-12, Zhengzhou Haiting Electronic Technology Co., Ltd.), plantar swelling tester (PV-200, Chengdu Taimeng Co., Ltd.), visible spectrophotometer (V-1100D, Shanghai Meipuda Instrument Co., Ltd.), intelligent hot plate (RB-200, Chengdu Taimeng Software Co., Ltd.), electronic tenderness instrument (YLS-3E, Beijing Zhongshidichuang Technology Development Co., Ltd.), and photothermal tail pain tester (SW-200, Chengdu Taimeng Software Co., Ltd.).

### 2.3 Experimental animals

The animals used in the study included 400 Kunming mice (SPF grade, 18–22 g, 225 females and 175 males) and 380 SD rats (SPF grade, 180–240 g, 130 females and 230 males). All mice and rats were provided by Chengdu Dashuo Experimental Animal Co., Ltd (licence numbers SCXK (Chuan) 2015-030, SCXK (Chuan) 2020-030; experimental animal quality certificate numbers: No. 51203500005170, No. 51203500008839, No. 51203500007444, No. 51203500007059, No. 51203500008739, No. 51203500009613, No. 51203500010565, No. 51203500009883, No. 51203500016746, No. 51203500005659, No. 51203500007266, No. 51203500007862, No. 51203500010612, No. 51203500010823, No. 51203500010613, and No. 51203500009311).

Animals were maintained in the Science and Technology Building at Wenjiang Campus (Chengdu University of Traditional Chinese Medicine, experimental animal use licence numbers SYXK (Chuan) 2014-124 and SYXK (Chuan) 2020-124). Experiments were conducted in the Laboratory of Ethnic Medicine Resource Evaluation at Chengdu University of Traditional Chinese Medicine (Third-Level Scientific Research Laboratory of the State Administration of Traditional Chinese Medicine, № TCM-2009-320).

### 2.4 UPLC-Q-TOF-MS/HPLC test conditions and sample preparation

#### 2.4.1 Equipment parameters and sample preparation of UPLC-Q-TOF-MS

Equipment parameters: For chromatography, the column was an ACQUITY BEH C18 column (2.1 mm × 150 mm, 1.7 μm), the mobile phases were acetonitrile solution (B)-0.1% formic acid aqueous solution (A) at a column temperature of 30°C, the flow rate was 0.4 mL min^–1^, and the injection volume was 2 μL. The gradient elution was 95%–20%, B at 0–20 min and 20%–5% B at 20–30 min. The mass spectrum conditions were as follows: electrospray ionization in the positive and negative modes, nitrogen flow rate of 600 L h^–1^, desolvation temperature of 350°C, capillary voltage of 3.0 kV, cone voltage of 30 kV, collision energy of 15–45 eV, ion source temperature of 120°C, and scanning range of m/z 50–1500.

Preparation of test and standard solutions: A volume of 10 mL of methanol was added to 0.1 g of ZXZTCs (four samples in parallel). Following ultrasonic treatment for 30 min, the liquid was passed through a 0.22 μm microporous filter and stored at 4°C as the test solution. Appropriate amounts of chlorogenic acid, forsythin B, shanzhiside methyl ester, 8-O acetyl shanzhiside methyl ester, verbascoside, loganin, and luteoloside were accurately weighed and placed in a 10 mL brown volumetric flask, dissolved in methanol, and diluted to the required volume to prepare a mixed standard solution with concentrations of 83, 104.75, 79.5, 115.13, 103.88, 75.25, and 101.38 μg/mL, respectively. The solution (2 mL) was passed through a 0.22 μm microporous filter and stored at 4°C as a mixed standard solution for testing.

Blood serum sample preparation: Twenty rats (10 male and 10 female) were divided into four batches (two batches of females and two batches of males, with five animals in each batch). Five rats were divided into two groups, specifically, ZXZTCs (four rats) and blank control (one rat). The ZXZTCs group was gavaged with the maximum dose (10.50 g/kg, 200 times the daily dose of 0.0525 g/kg for clinical adults) of ZXZTCs solution. For the maximum dose, ZXZTCs were dissolved in normal saline until the solution could be successfully extracted and administered to rats by gavage. At slightly higher concentrations, the solution could not be successfully gavaged. The blank control group was gavaged with the same dose of normal saline (20 mL/kg). At 30, 60, 90, and 120 min after gavage in the ZXZTCs group, blood was collected from the abdominal aorta of one animal at each time point. Blood was collected from the abdominal aorta directly after the gavage of normal saline in the blank control group. Subsequent batches were treated as above. After blood stasis for 10 min, the upper liquid layer was centrifuged at 3,500 r/min for 10 min. Blood sera were separated from both the blank control and ZXZTCs groups and stored at −80°C. During the test, 50 μL of the drug-containing serum solution at each of the four time points was precisely mixed to obtain 200 μL of drug-containing serum mixture, which was placed in a 2.0 mL EP tube. Next, 1,000 μL of acetonitrile precipitate was added, vortexed for 2 min for mixing, and centrifuged at 13,000 r/min and 4°C for 10 min. The supernatant was pipetted into a 2 mL EP tube, blow-dried at 37°C with a nitrogen blower, reconstituted with 200 μL of methanol, vortexed for 2 min, sonicated for 10 min, thoroughly mixed, and re-centrifuged at 13,000 r/min (4°C for 10 min). The collected supernatant was passed through a 0.22 μm microporous filter. An aliquot of blank serum (200 μL) was precisely pipetted into a 2.0 mL EP tube and processed in a similar manner as above.

Data processing and analysis: After UPLC-Q-TOF-MS analysis of the test solution, standard solution, blank serum, and drug-containing serum, the relevant mass spectrometric data were analyzed with MassLynx V4.2 software. The relative molecular mass of the compound was determined according to the quasi-molecular ion peak detected in the mass spectrum corresponding to the base peak ion flow chromatographic peak. Total metabolites and metabolites entered into the blood of ZXZTCs were identified by further comparison and speculation of cleavage fragment ion information from first- and second-stage mass spectra combined with data from the literature and standard solution experiments.

#### 2.4.2 Equipment parameters and sample preparation of HPLC

Equipment parameters: An Elite Supersil ODS2 chromatography column (250 × 4.6 mm^2^, 5 µm) was used with the following conditions: detection wavelength of 235 nm, the mobile phases were acetonitrile (B)−0.2% phosphoric acid water (C), column temperature of 30°C, flow rate of 1.0 mL min^–1^, and injection volume of 10 µL. The gradient elution procedure was as follows: 5%–6% B at 0–10 min, 6%–10% B at 10–25 min, 10%–13% B at 25–40 min, 13%–15% B at 40–50 min, and 15%–19% B at 50–80 min.

Preparation of test and standard solutions: ZXZTCs powder (0.5 g) was accurately weighed with a balance, placed in a 50 mL conical flask, and mixed with 25 mL of 70% methanol solution. The mixture was weighed, subjected to ultrasonic treatment (power, 200 W; frequency, 40 kHz) for 30 min, allowed to cool, and re-weighed with a balance. After the addition of 70% methanol to reduce weight loss, the mixture was shaken well. The filtrate was collected and subsequently passed through a 0.45 μm microporous filter membrane. The supernatant obtained was stored at 4°C as the test solution. Appropriate amounts of shanzhiside methyl ester, chlorogenic acid, 8-O acetyl shanzhiside methyl ester, forsythin B, luteoloside, and verbascoside chemicals were accurately weighed, placed in a 10 mL brown volumetric flask and dissolved in 10 mL methanol. After dilution to the required volume, the mixture was used to prepare a mixed standard solution with concentrations of 48.2, 64.9, 57.3, 172.8, 111, and 132 μg/mL, respectively, of the above six constituents, and stored as a mixed standard solution for testing.

### 2.5 Animal modeling, grouping, and dose design

For the evaluation of hemostasis, six animal experiments were conducted. In experiment 1, the models of bleeding time and coagulation time in mice were established (Yang et al., 2015; Xie et al., 2017). Mice were divided into blank control (normal saline), carbazochrome control (0.0025 g kg^–1^ d^–1^), low-dose ZXZTCs (0.2625 g kg^–1^ d^–1^), medium-dose ZXZTCs (0.5250 g kg^–1^ d^–1^), and high-dose ZXZTCs (1.0500 g kg^–1^ d^–1^) groups. The dosage volume was 20 mL/kg, with 10 animals per group (five male and five female) fed in separate cages. In experiment 2, the models of blood agglutination parameters and plasma recalcification time in rats were established (Li et al., 2006; Zhao et al., 2010). Rats were divided into blank control (normal saline), Yunnan Baiyao capsules control (Liu, 2012; Liao et al., 2014) (0.1667 g kg^–1^ d^–1^), low-dose ZXZTCs (0.1313 g kg^–1^ d^–1^), medium-dose ZXZTCs (0.2625 g kg^–1^ d^–1^), and high-dose ZXZTCs (0.5250 g kg^–1^ d^–1^) groups. The dosage volume was 10 mL/kg, with 10 animals per group (five male and five female) fed in separate cages. In experiment 3, the model of platelet aggregation rate in rats was established (Hideaki et al., 2017; Yin et al., 2018). Rats were divided into blank control (normal saline), Yunnan Baiyao capsules control (Shen et al., 2006; Shi et al., 2009; Shen et al., 2015; Luo, 2016) (0.1667 g kg^–1^ d^–1^), low-dose ZXZTCs (0.1313 g kg^–1^ d^–1^), medium-dose ZXZTCs (0.2625 g kg^–1^ d^–1^), and high-dose ZXZTCs (0.5250 g kg^–1^ d^–1^) groups. The dosage volume was 10 mL/kg, with 10 animals per group (five male and five female) fed in separate cages. In experiment 4, the model of thrombocytopenia induced by cytarabine in mice was established (Xu et al., 2005; Yoo et al., 2011; He et al., 2014). Mice were intraperitoneally injected with cytarabine (200 mg/kg) once a day for 2 consecutive days, which was switched to 50 mg/kg on day 3 once a day for 3 consecutive days at an injection volume of 20 mL/kg. Mice were divided into blank control (normal saline), model control (normal saline), prednisone acetate tablets control (Zhang et al., 2013; Zhao, 2017) (0.0100 g kg^–1^ d^–1^), low-dose ZXZTCs (0.2625 g kg^–1^ d^–1^), medium-dose ZXZTCs (0.5250 g kg^–1^ d^–1^), and high-dose ZXZTCs (1.0500 g kg^–1^ d^–1^) groups. The dosage volume was 20 mL/kg, with 10 animals per group (five male and five female) fed in separate cages. In experiment 5, the tail thrombosis model induced by carrageenan in mice was established (Simkhada et al., 2012; Yang et al., 2013) (1 h after the last administration, 0.1% carrageenan solution was injected intraperitoneally at a volume of 20 mL/kg). Mice were divided into model control (normal saline), aspirin enteric-coated tablets control (Ding, 2010) (0.05 g kg^–1^ d^–1^), low-dose ZXZTCs (0.2625 g kg^–1^ d^–1^), medium-dose ZXZTCs (0.5250 g kg^–1^ d^–1^), and high-dose ZXZTCs (1.0500 g kg^–1^ d^–1^) groups. The dosage volume was 20 mL/kg, with 10 animals per group (five male and five female) fed in separate cages. In experiment 6, the Chandler thrombus model *in vitro* of rats was established (Chen, 2004; Jiang et al., 2004; Wang H. et al., 2010; McClung et al., 2010; Stefanie et al., 2013; Song, 2012; Gaammangwe et al., 2014; Hisham et al., 2014; Yu et al., 2015). Rats were divided into model control (normal saline), aspirin enteric-coated tablets control (Ma et al., 2002; Gao et al., 2005; Yu, 2007; Yang, 2008; Chen et al., 2013) (0.025 g kg^–1^ d^–1^), low-dose ZXZTCs (0.1313 g kg^–1^ d^–1^), medium-dose ZXZTCs (0.2625 g kg^–1^ d^–1^), and high-dose ZXZTCs (0.5250 g kg^–1^ d^–1^) groups. The dosage volume was 10 mL/kg, with 10 animals per group (five male and five female) fed in separate cages. The treatments were administered by gavage once a day for 7 days.

For the evaluation of analgesia, five animal experiments were conducted. In experiment 1, the writhing reaction model induced by acetic acid in mice was established (Ribeiro et al., 2000; Olufunmilayo et al., 2008; Wang et al., 2008; Wu et al., 2015; Wang. et al., 2017; Xu et al., 2017) (1 h after the final administration, each mouse was injected intraperitoneally with 0.6% acetic acid at a dose of 10 mL/kg). Mice were divided into model control (normal saline), aspirin enteric-coated tablets control (Yang et al., 2007; Wang, 2013; Li. et al., 2016) (0.05 g kg^–1^ d^–1^), low-dose ZXZTCs (0.2625 g kg^–1^ d^–1^), medium-dose ZXZTCs (0.5250 g kg^–1^ d^–1^), and high-dose ZXZTCs (1.0500 g kg^–1^ d^–1^) groups. The dosage volume was 20 mL/kg, with 10 animals per group (five male and five female) fed in separate cages. In experiment 2, the hot plate-induced foot pain model in mice was established (Huang et al., 2000; Luo et al., 2008; Su et al., 2011; Wu et al., 2012; Yin et al., 2016). Mice were divided into model control (normal saline), aspirin enteric-coated tablets control (Zhang et al., 2008a; Huo et al., 2008; Qian et al., 2012; Wan et al., 2013) (0.05 g kg^–1^ d^–1^), low-dose ZXZTCs (0.2625 g kg^–1^ d^–1^), medium-dose ZXZTCs (0.5250 g kg^–1^ d^–1^), and high-dose ZXZTCs (1.0500 g kg^–1^·d^–1^) groups. The dosage volume was 20 mL/kg, with 10 animals per group (all females) maintained in a single cage. In experiment 3, the foot tenderness model induced by mechanical stimulation in rats was established (Célèrier et al., 1999; Wang. et al., 2017; Li et al., 2017; Pang et al., 2018). Rats were divided into model control (normal saline), rotundine tablets control (Wang. et al., 2010) (0.030 g kg^–1^ d^–1^), low-dose ZXZTCs (0.1313 g kg^–1^ d^–1^), medium-dose ZXZTCs (0.2625 g kg^–1^ d^–1^), and high-dose ZXZTCs (0.5250 g kg^–1^ d^–1^) groups. The dosage volume was 10 mL/kg, with 10 animals per group (five male and five female) fed in separate cages. In experiment 4, the tail flick reaction model induced by photothermal stimulation in rats was established (Xu et al., 1994; Zhang et al., 2002; Zhang et al., 2012; Lim et al., 2014; Liu et al., 2015; Yohannes et al., 2017). Rats were divided into model control (normal saline), rotundine tablets control (Wang. et al., 2010) (0.030 g kg^–1^ d^–1^), low-dose ZXZTCs (0.1313 g kg^–1^ d^–1^), medium-dose ZXZTCs (0.2625 g kg^–1^ d^–1^), and high-dose ZXZTCs (0.5250 g kg^–1^ d^–1^) groups. The dosage volume was 10 mL/kg, with 10 animals per group (five male and five female) fed in separate cages. In experiment 5, the dysmenorrhea model induced by oxytocin in mice was established (Sun et al., 2002; Wang et al., 2002; Bai, 2005; Gao et al., 2011; Li. et al., 2012; Li. et al., 2012; Cao et al., 2012; Su et al., 2012; Sun et al., 2013; Amol et al., 2017; Suhas et al., 2018). The primary dysmenorrhea model was generated with estrogen and oxytocin and estradiol valerate administered by continuous gavage (2 mg/kg on day 1, 1 mg/kg on days 2–9, 2 mg/kg on day 10, and oxytocin 20 U/kg was injected intraperitoneally on day 11 until the animal showed a writhing response). Mice were divided into model control (normal saline), ibuprofen capsules control (Li et al., 2016) (0.1333 g kg^–1^·d^–1^), low-dose ZXZTCs (0.2625 g kg^–1^ d^–1^), medium-dose ZXZTCs (0.5250 g kg^–1^ d^–1^), and high-dose ZXZTCs (1.0500 g kg^–1^ d^–1^) groups. The dose volume was 20 mL/kg, with 10 animals per group (all females) maintained in a single cage. The above treatments were administered by gavage once a day for 7 days.

For the evaluation of anti-inflammatory activity, four animal experiments were conducted. In experiment 1, the ear swelling model induced by xylene in mice was established (Gu et al., 2016) (1 h after the final administration, 50 µL of xylene was applied evenly on the inside and outside of the right ear contour of each mouse). Mice were divided into model control (normal saline), prednisone acetate control (Feng et al., 2013) (0.01 g kg^–1^ d^–1^), low-dose ZXZTCs (0.2625 g kg^–1^ d^–1^), medium-dose ZXZTCs (0.5250 g kg^–1^ d^–1^), and high-dose ZXZTCs (1.0500 g kg^–1^ d^–1^) groups. The dosage volume was 20 mL/kg, with 10 animals per group (five male and five female) fed in separate cages. In experiment 2, the foot swelling model induced by carrageenan in rats was established (Yan et al., 2004; Wang. et al., 2018) (1 h after the final administration, 0.1 mL of 1% carrageenan was injected into the foot pad of the right hind leg of rats in each group to induce inflammation). Rats were divided into model control (normal saline), prednisone acetate control (Lin et al., 2010) (0.005 g kg^–1^ d^–1^), low-dose ZXZTCs (0.1313 g kg^–1^ d^–1^), medium-dose ZXZTCs (0.2625 g kg^–1^ d^–1^), and high-dose ZXZTCs (0.5250 g kg^–1^ d^–1^) groups. The dosage volume was 10 mL/kg, with 10 animals per group (all males) maintained in separate cages. In experiment 3, the model of peritoneal permeability induced by acetic acid in mice was established (Feng et al., 2013; Ouyang et al., 2014) (1 h after the final administration, the mice in each group were injected with 1% Evans blue normal saline solution through the tail vein according to standard (0.1 mL/10 g) body weight, followed by immediate intraperitoneal injection of 0.2 mL of 0.6% acetic acid). Mice were divided into model control (normal saline), chlorphenamine maleate tablets control (Zhang et al., 2016) (0.002 g kg^–1^ d^–1^), low-dose ZXZTCs (0.2625 g kg^–1^ d^–1^), medium-dose ZXZTCs (0.5250 g kg^–1^ d^–1^), and high-dose ZXZTCs (1.0500 g kg^–1^ d^–1^) groups. The dosage volume was 20 mL/kg, with 10 animals per group (all males) maintained in a single cage. In experiment 4, the cotton ball-induced granuloma model in rats was established (Yang et al., 2009; Efimova et al., 2010; Zelenin et al., 2011; Wang et al., 2016) (rats were anesthetised via intraperitoneal injection of 20% urethane solution (0.6 mL/100 g), a 0.5 cm incision made in the abdomen, and a 20 mg sterile cotton ball implanted under the skin of the right groin, following which the incision was sutured and disinfected). Rats were divided into model control (normal saline), prednisone acetate control (Ma et al., 2016) (0.005 g kg^–1^ d^–1^), low-dose ZXZTCs (0.1313 g kg^–1^ d^–1^), medium-dose ZXZTCs (0.2625 g kg^–1^ d^–1^), and high-dose ZXZTCs (0.5250 g kg^–1^ d^–1^) groups. The dosage volume was 10 mL/kg, with 10 animals per group (all males) maintained in separate cages. Treatments were administered by gavage once a day for 7 days.

In mice experiments, the low, medium, and high doses of ZXZTCs were used at 5, 10, and 20 times of the adult daily dose (0.0525 g/kg) and the positive control dose was 10 times that of the adult daily dose.

In rat experiments, the low, medium, and high doses of ZXZTCs were used 2.5, 5, and 10 times of the adult daily dose (0.0525 g/kg) and the positive control dose was 5 times that of the adult daily dose.

### 2.6 Anesthetisation and handling of animals

After samples were collected or the relevant indicators were tested, animals were anesthetized via intraperitoneal injection of 20% urethane solution (0.6 mL/100 g) and sacrificed through excessive blood loss from the abdominal aorta.

### 2.7 Statistical analysis

Data are expressed as mean ± standard deviation ( $\bar{x}\pm s$ ). The independent sample *t*-test or one-way ANOVA provided by SPSS 17.0 for Windows software was employed to assess the significance of mean differences between two or more groups of data. Levene’s test for homogeneity of variance was performed. At *p* values > 0.05, variance was homogeneous. *p* values were additionally determined using a *t*-test or least significant difference test. At *p* values < 0.05, variance was inhomogeneous. The statistical significance of data was further analyzed with a corresponding *t*-test or Tamhane’s T2 test. The ranked data were obtained using a non-parametric Mann-Whitney test with SPSS 17.0 for Windows.

## 3 Results

### 3.1 Qualitative analysis of metabolites in ZXZTCs via UPLC-Q-TOF-MS

**FIGURE 1:**
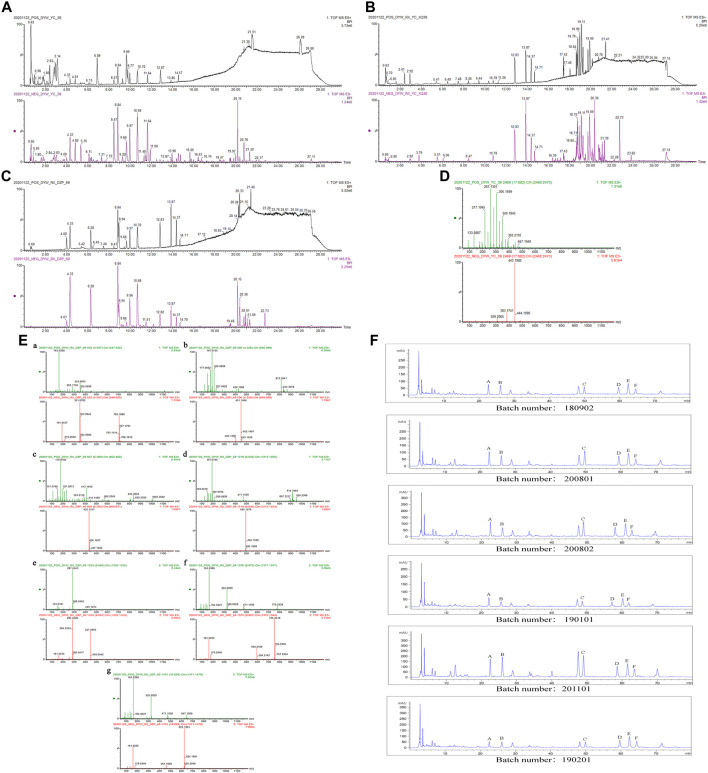
**(A)** BPI diagram of UPLC-Q-TOF-MS of ZXZTCs. **(B)** BPI diagram of drug-containing serum UPLC-Q-TOF-MS. **(C)** BPI diagram of UPLC-Q-TOF-MS of mixed standard substance (4.01, chlorogenic acid; 4.33, shanzhiside methyl ester; 6.28, loganin; 8.84, 8-O-acetyl shanzhiside methyl ester; 9.66, luteoloside; 9.97, forsythin B; 10.68, verbascoside). **(D)** Mass spectrum of 6β-n-butoxy-7,8-dehydropentemonoside. **(E)** UPLC-Q-TOF-MS mass spectrum of each standard substance. a. Chlorogenic acid. b. Shanzhiside methyl ester. c. Loganin. d. 8-O-acetyl shanzhiside methyl ester. e. Luteoloside. f. Forsythin B. g. Verbascoside. **(F)** HPLC content determination of each batch of ZXZTCs (Note: A, shanzhiside methyl ester; B, chlorogenic acid; C, 8-O-acetyl shanzhiside methyl ester; D, forsythin B; E, luteoloside; F, verbascoside).

ZXZTCs were analyzed by UPLC-Q-TOF-MS in both positive and negative ion modes. According to the standard substance atlas (Figures 1C, E) and published metabolite data for *L. rotata* (Yi et al., 1992; Afifi et al., 1993; Li et al., 1999; Tripetch et al., 2002; Zhang, 2008b; Xue et al., 2009; Wu et al., 2013; Pan, 2015; Wu et al., 2016; Pan et al., 2018; Wang et al., 2018; Zan et al., 2018), a total of 36 metabolites from 5 categories were detected in the base peak ion (BPI) chromatogram of ZXZTCs (Figure 1A). By comparing the retention times and relative molecular masses of these metabolites with the standard substance (Figure 1) and references, we deduced the presence of 13 iridoid glycosides, nine flavonoids, nine phenylethanol glycosides, four phenylpropanoids, and one other metabolite (Table 1).

**TABLE 1:** MS data of metabolites of ZXZTCs.

Number	t_R_ (min)	Chemical formula	Molecular mass	ESI^-^ and its fragment ions	ESI^+^ and its fragment ions	Chemical name	Classification	References
1	1.62	C_17_H_26_O_13_	438	437	461	Phloyoside I^b^	Iridoid glycoside	Pan (2015)
				483	181			
				419				
2	1.89	C_16_H_24_O_11_	392	391	——	Shanzhiside^b^	Iridoid glycoside	Wu et al. (2016)
3	2.83	C_17_H_24_O_11_	404	449	427	Phlorigidoside C^b^	Iridoid glycoside	Pan, 2015; Zan et al. (2018)
4	3.43	C_17_H_24_O_12_	420	419	443	Sesamoside^b^	Iridoid glycoside	Pan (2015)
				465	863			
5	3.67	C_11_H_14_O_7_	258	257	539	Lamiophlomiol C^b^	Iridoid glycoside	Yi et al. (1992), Wu et al. (2016)
				515				
6	3.79	C_25_H_38_O_16_	594	593	612	Markhamioside A^b^	Phenylpropanoid	Tripetch et al. (2002), Pan et al. (2018)
					649			
7	4	C_16_H_18_O_9_	354	353	355	Chlorogenic acid^ab^	Phenylpropanoid	Zan et al. (2018)
				707	283			
8	4.33	C_17_H_26_O_11_	406	405	813	Shanzhiside methyl ester^ab^	Iridoid glycoside	La et al., 2015; Pan (2015), Wu et al. (2016)
				451	429			
					209			
9	4.82	C_16_H_24_O_10_	376	375	775	Loganic acid^b^	iridoid glycoside	Wu et al. (2016)
				751	399			
					179			
10	5.35	C_17_H_25_O_12_Cl	456	455	935	Phloyoside Ⅲ^b^	Iridoid glycoside	Pan (2015)
				501	479			
				911	277			
11	6.12	C_17_H_25_ClO_11_	440	439	934	Chlorotuberoside^b^	Iridoid glycoside	La et al. (2015), Xue et al. (2009)
				485	495			
					278			
12	7.21	C_34_H_44_O_20_	772	771	——	3,4-Dihydroxyphenylethanol-8-O-[4-O-transcaffeoyl-β-d-apiofuranosyl(1→3)-β-d-glucopyranosyl-(1→6)]-β-d-glucopyranoside^b^	Flavonoid	Wang et al. (2018a)
				795				
13	7.31	C_17_H_26_O_10_	390	435	413	Loganin^ab^	Iridoid glycoside	Pan (2015)
					211			
14	7.51	C_29_H_36_O_16_	640	639	663	Campneoside II^b^	Phenylethanol glycoside	La et al. (2015)
15	7.72	C_21_H_20_O_12_	464	463	465	Hyperoside^b^	Flavonoid	Wu et al. (2016)
16	8.47	C_16_H_24_O_9_	360	359	743	7-Deoxyloganic acid^b^	Iridoid glycoside	Wu et al. (2016)
				719	383			
17	8.84	C_19_H_28_O_12_	448	493	919	8-O-acetyl shanzhiside methyl ester^ab^	Iridoid glycoside	Pan (2015)
					471			
					191			
18	9.29	C_26_H_28_O_15_	580	579	581	Luteolin-7-O-β-D-apiofuranosyl (1→6)-O-β-D-glucopyranoside^b^	Flavonoid	Wu et al. (2016), Zan et al. (2018)
19	9.66	C_21_H_20_O_11_	448	447	449	Luteoloside^ab^	Flavonoid	Pan, 2015; Zan et al. (2018)
				895	287			
20	9.97	C_34_H_44_O_19_	756	755	779	Forsythin B^ab^	Phenylethanol glycoside	Pan (2015)
21	10.69	C_29_H_36_O_15_	624	623	647	Verbascoside^ab^	Phenylethanol glycoside	Pan, 2015; Zan et al. (2018)
22	11.15	C_26_H_28_O_14_	564	563	565	Apigenin 7-O-β-D-apiofuranosyl (1→6)-β-D-glucopyranoside	Flavonoid	La et al. (2015)
					587			
23	11.64	C_29_H_36_O_15_	624	623	625	Isoacteoside^b^	Phenylethanol glycoside	Zan et al. (2018)
					647			
24	11.9	C_35_H_46_O_19_	770	769	793	6-β-D-Apioufarnosyl cistanoside C	Phenylpropanoid	Pan (2015)
25	13.13	C_30_H_38_O_15_	638	637	661	Alyssonoside^b^	Phenylpropanoid	Pan (2015)
26	13.64	C_36_H_48_O_19_	784	783	802	Lamiophlomioside A^b^	Phenylethanol glycoside	Wu et al. (2016)
				829	807			
27	13.97	C_36_H_48_O_19_	784	783	807	Leucosceptoside B^b^	Phenylethanol glycoside	Pan (2015)
28	14.39	C_23_H_22_O_12_	490	489	491	Luteolin-7-O-β-D-(6"-O-acetate)-glucopyranoside^b^	Flavonoid	La et al. (2015)
29	14.57	C_15_H_10_O_6_	286	285	287	Luteolin^b^	Flavonoid	Zan et al. (2018)
30	14.75	C_20_H_30_O_12_	462	461	485	Decaffeoyl acteoside^b^	Phenylethanol glycoside	Li et al. (1999), Pan et al. (2018)
				507	445			
31	15.55	C_30_H_26_O_12_	578	577	1157	Apigenin-7-O-β-D- (-6"-p-coumaroyl)-glucoside)	Flavonoid	Zhang (2008b)
32	15.65	C_28_H_36_O_16_	628	627	629	6-O-syringyl-barlerin^b^	Iridoid glycoside	La et al. (2015)
					651			
					569			
33	17.88	C_21_H_32_O_10_	444	443	445	6β-n-butoxy-7,8- dehydropenstemonoside^b^	Other	La et al. (2015)
					467			
34	18.1	C_15_H_10_O_5_	270	269	——	Apigenin^b^	Flavonoid	Zan et al. (2018)
35	19.79	C_30_H_36_O_14_	620	619	621	Rossicaside C^b^	Phenylethanol glycoside	Wang et al. (2018a), Wu et al. (2013)
					643			
					603			
36	22.17	C_29_H_34_O_15_	622	621	——	Crenatoside^b^	Phenylethanol glycoside	Wang et al. (2018a), Afifi et al. (1993)

^a^ Indicates a comparison with the standard substance.

^b^ Indicates the metabolites have been reported previously in *L. rotata*.

#### 3.1.1 Representative metabolites of iridoid glycosides

**FIGURE 2:**
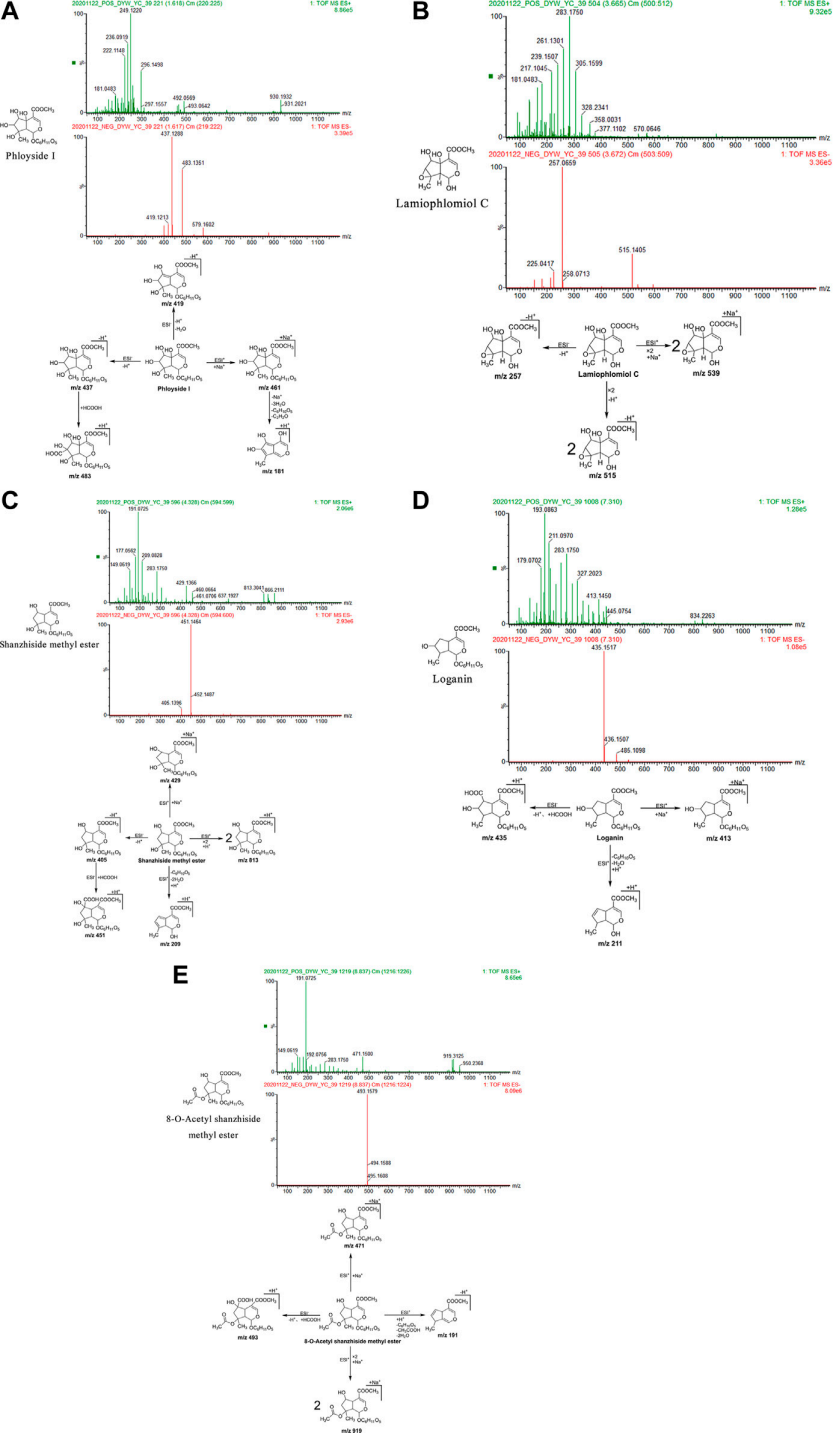
Structural formulas, mass spectra, and fragmentation processes of representative metabolites of iridoid glycosides. **(A)** Phloyoside I. **(B)** Lamiophlomiol C. **(C)** Shanzhiside methyl ester. **(D)** Loganin. **(E)** 8-O-acetyl shanzhiside methyl ester.

Phloyoside I: The retention time in the UPLC system was 1.62 min. In the ESI negative ion mode, a quasi-molecular ion peak at m/z 437 [M-H]^–^ was obtained. Molecular ion peaks at m/z 483 [M-H + HCOOH]^–^ and 419 [M-H-H_2_O]^–^ were additionally detected. In the ESI positive ion mode, a molecular ion peak at m/z 461 [M + Na]+ appeared, along with fragment ions at m/z 181 [M + H-3H_2_O-C_6_H_10_O_5_-C_2_H_2_O]^+^. The molecular mass of the metabolite was confirmed as 438. Based on the present data in combination with previous literature (Pan, 2015), the identity of the metabolite was deduced as phloyoside I with a molecular formula of C_17_H_26_O_13_. The mass spectrum, structural formula, and fragmentation process are shown in Figure 2A.

Lamiophlomiol C: The retention time in the UPLC system was 3.67 min. In the ESI negative ion mode, a quasi-molecular ion peak at m/z 257 [M-H]– was obtained. Additionally, a molecular ion peak at m/z 515 [2M-H]^–^ appeared in the spectrum. In the ESI positive ion mode, a molecular ion peak at m/z 539 [2M + Na]^+^ was detected. The molecular mass of the metabolite was confirmed as 258. Based on the present data in combination with earlier literature (Wu et al., 2016), the identity of the metabolite was deduced as lamiophlomiol C with a molecular formula of C_11_H_14_O_7_. The mass spectrum, structural formula (Yi et al., 1992), and fragmentation process are shown in Figure 2B.

Shanzhiside methyl ester: The retention time in the UPLC system was 4.33 min. In the ESI negative ion mode, a quasi-molecular ion peak at m/z 405 [M-H]^–^ was obtained. Additionally, a fragment ion of a molecular ion peak at m/z 451 [M-H + HCOOH]^–^ appeared in the spectrum. In the ESI positive ion mode, molecular ion peaks at m/z 813 [2M + H]+and 429 [M + Na]+were detected, along with fragment ions at m/z 209 [M + H-C_6_H_10_O_5_-2H_2_O]^+^. The molecular mass of the metabolite was confirmed as 406. Combined with earlier literature (La et al., 2015; Pan, 2015; Wu et al., 2016) and data from comparisons with the standard substance, our findings inferred that the metabolite was shanzhiside methyl ester with a molecular formula of C_17_H_26_O_11_. The mass spectrum, structural formula (Pan, 2015) and fragmentation process are shown in Figure 2C. The mass spectrum of the standard substance is shown in Figure 1.

Loganin: The retention time in the UPLC system was 7.31 min. In the ESI negative ion mode, fragment ions of a molecular ion peak at m/z 435 [M-H + HCOOH]^–^ were obtained. In the ESI positive ion mode, a molecular ion peak at m/z 413 [M + Na]^+^ was detected. Moreover, fragment ions at m/z 211 [M + H-C_6_H_10_O_5_-H_2_O]^+^ appeared. The molecular mass of the metabolite was determined as 390. Combined with earlier literature (Pan, 2015) and data from a comparison with the standard substance, our findings inferred that the metabolite was loganin with a molecular formula of C_17_H_26_O_10_. The mass spectrum, structural formula (Pan, 2015), and fragmentation process are shown in Figure 2D. The mass spectrum of the standard substance is shown in Figure 1.

8-O-acetyl shanzhiside methyl ester: The retention time in the UPLC system was 8.84 min. In the ESI negative ion mode, a molecular ion peak at m/z 493 [M-H + HCOOH]^–^ was obtained. In the ESI positive ion mode, molecular ion peaks at m/z 919 [2M + Na]+and 471 [M + Na]^+^ were detected, along with fragment ions at m/z 191 [M + H-C_6_H_10_O_5_-CH_3_COOH-2H_2_O] ^+^. The molecular mass of the metabolite was confirmed as 448. Combined with earlier literature (Pan, 2015) and data from a comparison with the standard substance, our findings inferred that the metabolite was 8-O-acetyl shanzhiside methyl ester with a molecular formula of C_19_H_28_O_12_. The mass spectrum, structural formula (Pan, 2015), and fragmentation process are shown in Figure 2E. The mass spectrum of the standard substance is shown in Figure 1.

#### 3.1.2 Representative metabolites of flavonoids

**FIGURE 3:**
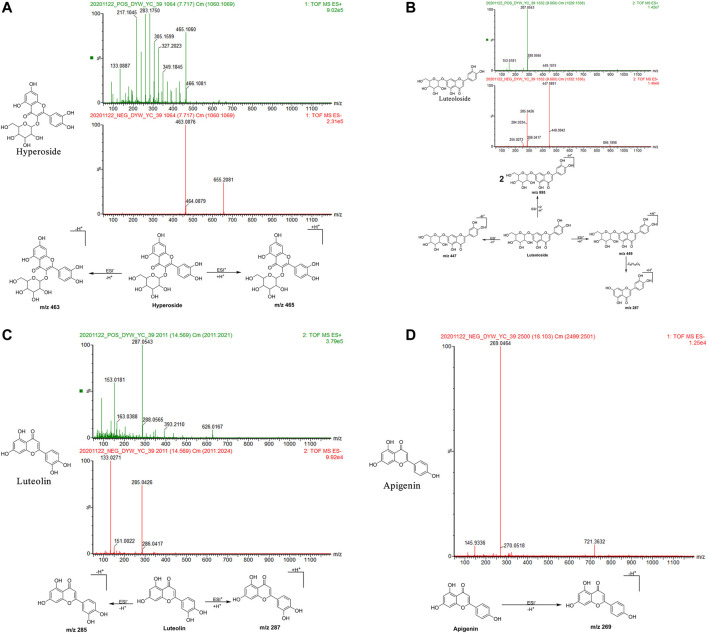
Structural formulas, mass spectra, and fragmentation processes of representative metabolites of flavonoids. **(A)** Hyperoside. **(B)** Luteoloside. **(C)** Luteolin. **(D)** Apigenin.

Hyperoside: The retention time in the UPLC system was 7.72 min. In the ESI negative ion mode, a quasi-molecular ion peak at m/z 463 [M-H]^–^ was obtained. In the ESI positive ion mode, a quasi-molecular ion peak at m/z 465 [M + H]^+^was obtained. The molecular mass of the metabolite was confirmed as 464. Based on the present data in combination with earlier literature (Wu et al., 2016), the identity of the metabolite was deduced as hyperoside with a molecular formula of C_21_H_20_O_12_. The mass spectrum, structural formula, and fragmentation process are shown in Figure 3A.

Luteoloside: The retention time in the UPLC system was 9.66 min. In the ESI negative ion mode, a quasi-molecular ion peak at m/z 447 [M-H]^–^ and molecular ion peak at m/z 895 [2M-H]^–^ were obtained. In the ESI positive ion mode, a quasi-molecular ion peak at m/z 449 [M + H]^+^was detected, along with fragment ions of a molecular ion peak at m/z 287 [M + H-C_6_H_10_O_5_]^+^. The molecular mass of the metabolite was confirmed as 448. Combined with earlier literature (Pan, 2015; Zan et al., 2018) and data from comparisons with the standard substance, the identity of the metabolite was inferred as luteoloside with a molecular formula of C_21_H_20_O_11_. The mass spectrum, structural formula (Pan, 2015), and fragmentation process are shown in Figure 3B. The mass spectrum of the standard substance is shown in Figure 1.

Luteolin: The retention time in the UPLC system was 14.57 min. In the ESI negative ion mode, a quasi-molecular ion peak at m/z 285 [M-H]^–^ was obtained. In the ESI positive ion mode, a quasi-molecular ion peak at m/z 287 [M + H]^+^appeared. The molecular mass of the metabolite was confirmed as 286. Based on the present data in combination with earlier literature (Zan et al., 2018), the identity of the metabolite was inferred as luteolin with a molecular formula of C_15_H_10_O_6_. The mass spectrum, structural formula, and fragmentation process are shown in Figure 3C.

Apigenin: The retention time in the UPLC system was 18.10 min. In the ESI negative ion mode, a quasi-molecular ion peak at m/z 269 [M-H]^–^ was obtained. The molecular mass of the metabolite was confirmed as 270. Based on the present data in combination with earlier literature (Zan et al., 2018), the identity of the metabolite was inferred as apigenin with a molecular formula of C_15_H_10_O_5_. The mass spectrum, structural formula, and fragmentation process are shown in Figure 3D.

#### 3.1.3 Representative metabolites of phenylethanol glycosides

**FIGURE 4:**
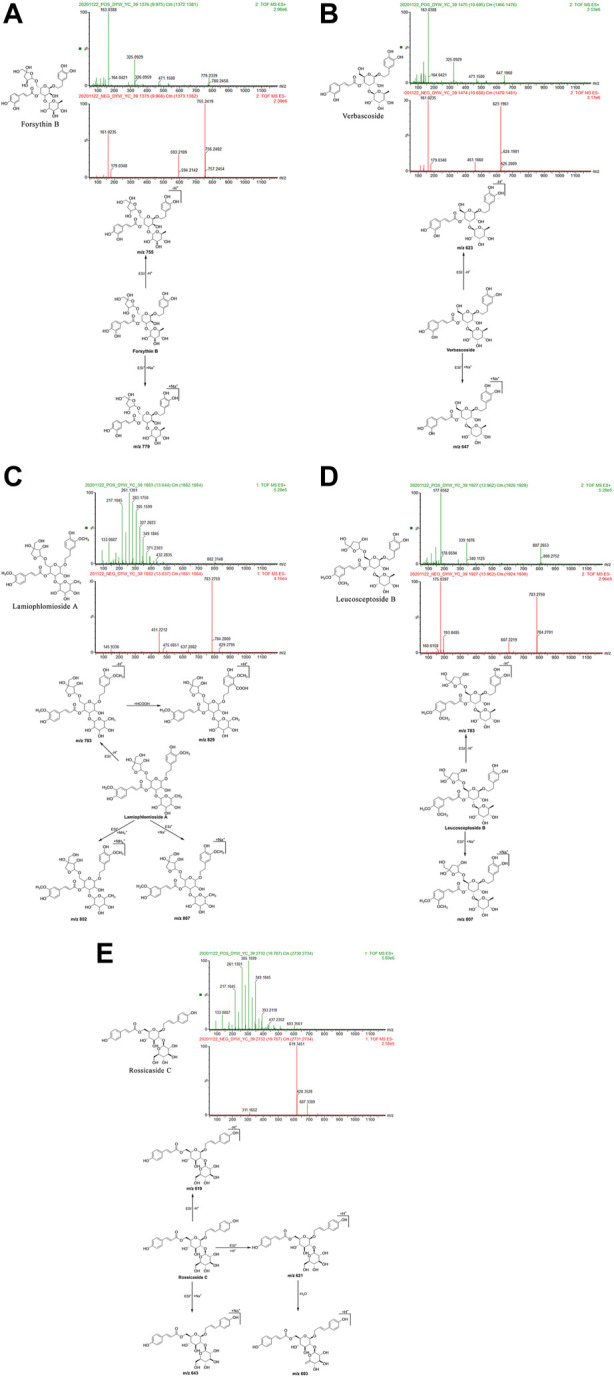
Structural formulas, mass spectra, and fragmentation processes of representative metabolites of phenylethanol glycosides. **(A)** Forsythin B. **(B)** Verbascoside. **(C)** Lamiophlomioside A. **(D)** Leucosceptoside B. **(E)** Rossicaside C.

Forsythin B: The retention time in the UPLC system was 9.97 min. In the ESI negative ion mode, a quasi-molecular ion at peak m/z 755 [M-H]^–^ was obtained. In the ESI positive ion mode, a molecular ion peak at m/z 779 [M + Na]^+^was obtained. The molecular mass of the metabolite was confirmed as 756. Combined with earlier literature (Pan, 2015) and data from a comparison with the standard substance, the identity of the metabolite was deduced as forsythin B with a molecular formula of C_34_H_44_O_19_. The mass spectrum, structural formula (Pan, 2015), and fragmentation process are shown in Figure 4A. The mass spectrum of the standard substance is shown in Figure 1.

Verbascoside: The retention time in the UPLC system was 10.69 min. In the ESI negative ion mode, a quasi-molecular ion peak at m/z 623 [M-H]^–^ was obtained. In the ESI positive ion mode, a molecular ion peak at m/z 647 [M + Na]^+^was detected. The molecular mass of the metabolite was confirmed as 624. Combined with earlier literature (Pan, 2015; Zan et al., 2018) and data from comparisons with the standard substance, the identity of the metabolite was deduced as verbascoside with a molecular formula of C_29_H_36_O_15_. The mass spectrum, structural formula (Pan, 2015), and fragmentation process are shown in Figure 4B. The mass spectrum of the standard substance is shown in Figure 1.

Lamiophlomioside A: The retention time in the UPLC system was 13.64 min. In the ESI negative ion mode, a quasi-molecular ion peak at m/z 783 [M-H]^–^ was obtained, in addition to a molecular ion peak at m/z 829 [M-H + HCOOH]^+^. In the ESI positive ion mode, molecular ion peaks at m/z 802 [M + NH_4_]^+^and 807 [M + Na]^+^were detected. The molecular mass of the metabolite was confirmed as 784. In combination with earlier literature (Wu et al., 2016), we inferred that the metabolite was lamiophnomioside A with a molecular formula of C_36_H_48_O_19_. The mass spectrum, structural formula, and fragmentation process are shown in Figure 4C.

Leucosceptoside B: The retention time in the UPLC system was 13.97 min. In the ESI negative ion mode, a quasi-molecular ion peak at m/z 783 [M-H]^–^ was obtained. In the ESI positive ion mode, a molecular ion peak at m/z 807 [M + Na]^+^was obtained. The molecular mass of the metabolite was confirmed as 784. In combination with earlier literature (Pan, 2015), our findings inferred that the metabolite was leucosceptoside B with a molecular formula of C_36_H_48_O_19_. The mass spectrum, structural formula, and fragmentation process are shown in Figure 4D.

Rossicaside C: The retention time in the UPLC system was 19.79 min. In the ESI negative ion mode, a quasi-molecular ion peak at m/z 619 [M-H]^–^ was obtained. In the ESI positive ion mode, a quasi-molecular ion peak at m/z 621 [M + H]^+^was obtained. In addition, a molecular ion peak at m/z 643 [M + Na]^+^and another fragment ion at m/z 603 [M + H-H_2_O]^+^appeared in the spectrum. The molecular mass of the metabolite was confirmed as 620. In combination with earlier literature (Wang et al., 2018), our findings inferred that the metabolite was rossicaside C with a molecular formula of C_30_H_36_O_14_. The mass spectrum, structural formula (Wu et al., 2013), and fragmentation process are shown in Figure 4E.

#### 3.1.4 Representative metabolites of phenylpropanoids

**FIGURE 5:**
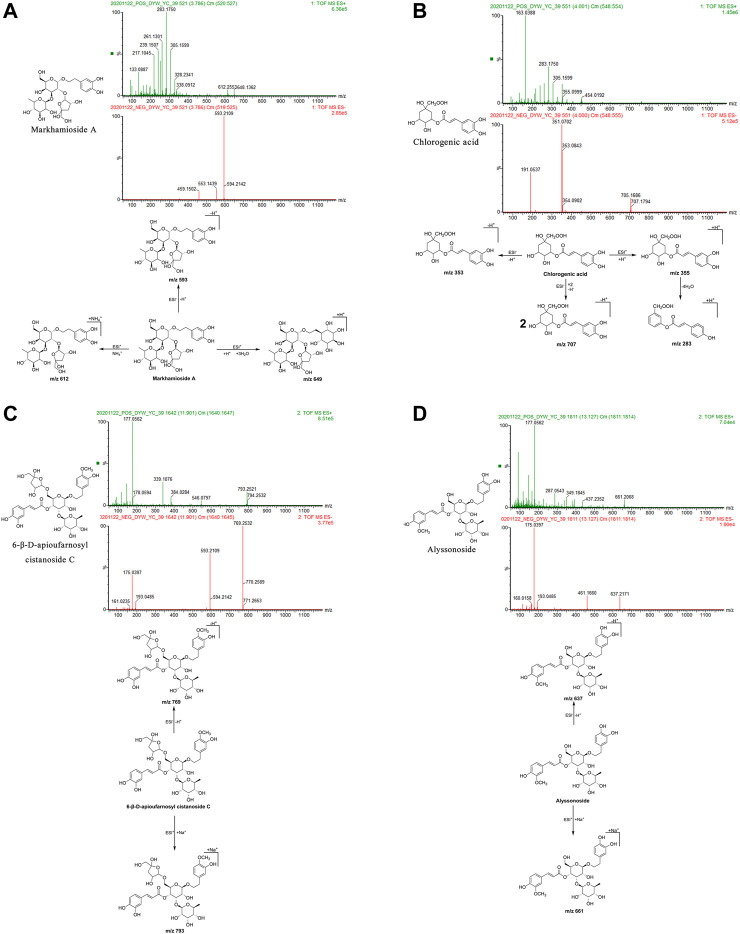
Structural formulas, mass spectra, and fragmentation processes of representative metabolites of phenylpropanoids. **(A)** Markhamioside A. **(B)** Chlorogenic acid. **(C)** 6-β-D-apioufarnosyl cistanoside C. **(D)** Alyssonoside.

Markhamioside A: The retention time in the UPLC system was 3.79 min. In the ESI negative ion mode, a quasi-molecular ion peak at m/z 593 [M-H]^–^ was obtained. In the ESI positive ion mode, molecular ion peaks at m/z 612 [M + NH_4_]^+^and 649 [M + H+3H_2_O]^+^were obtained. The molecular mass of the metabolite was confirmed as 594. Based on the present data in combination with earlier literature (Pan et al., 2018), the identity of the metabolite was deduced as markhamioside A with a molecular formula of C_25_H_38_O_16_. The mass spectrum, structural formula (Xue et al., 2009), and fragmentation process are shown in Figure 5A.

Chlorogenic acid: The retention time in the UPLC system was 4.00 min. In the ESI negative ion mode, a quasi-molecular ion peak at m/z 353 [M-H]^–^ and a molecular ion peak at m/z 707 [2M-H]^–^ appeared in the spectrum. In the ESI positive ion mode, a quasi-molecular ion peak at m/z 355 [M + H]^+^was obtained. Fragment ions of m/z 283 [M + H-4H_2_O]^+^were additionally detected. The molecular mass of the metabolite was confirmed as 354. Combined with earlier literature (Zan et al., 2018) and data from a comparison with the standard substance, our findings inferred that the metabolite was chlorogenic acid with a molecular formula of C_16_H_18_O_9_. The mass spectrum, structural formula, and fragmentation process are presented in Figure 5B. The mass spectrum of the standard substance is shown in Figure 1.

6-β-d-Apioufarnosyl cistanoside C: The retention time in the UPLC system was 11.90 min. In the ESI negative ion mode, a quasi-molecular ion peak at m/z 769 [M-H]^–^ was obtained. In the ESI positive ion mode, a molecular ion peak at m/z 793 [M + Na]^+^appeared. The molecular mass of the metabolite was confirmed as 770. In combination with earlier literature (Pan, 2015), our findings inferred that the metabolite was 6-β-d-apioufarnosyl cistanoside C with a molecular formula of C_35_H_46_O_19_. The mass spectrum, structural formula (Pan, 2015), and fragmentation process are shown in Figure 5C.

Alyssonoside: The retention time in the UPLC system was 13.13 min. In the ESI negative ion mode, a quasi-molecular ion peak at m/z 637 [M-H]^–^ was obtained. In the ESI positive ion mode, a molecular ion peak at m/z 661 [M + Na]^+^was obtained. The molecular mass of the metabolite was confirmed as 638. In combination with earlier literature (Pan, 2015), our findings inferred that the metabolite was alyssonoside with a molecular formula of C_30_H_38_O_15_. The mass spectrum, structural formula (Pan, 2015), and fragmentation process are shown in Figure 5D.

#### 3.1.5 Other metabolites

6β-n-butoxy-7,8-dehydropenstemonoside: The retention time in the UPLC system was 17.88 min. In the ESI negative ion mode, a quasi-molecular ion peak at m/z 443 [M-H]^–^ was obtained. In the ESI positive ion mode, a quasi-molecular ion peak at m/z 445 [M + H]^+^and a molecular ion peak at m/z 467 [M + Na]^+^were detected. The molecular mass of the metabolite was confirmed as 444. In combination with earlier literature (La et al., 2015), we inferred that the metabolite was 6β-n-butoxy-7,8-dehydropentemonoside with a molecular formula of C_21_H_32_O_10_. The mass spectrum is shown in Figure 1D.

### 3.2 Qualitative UPLC-Q-TOF-MS analysis of ZXZTCs metabolites that entered the blood

A total of 11 metabolites from three categories were detected in the base peak ion (BPI) chromatogram of the drug-containing serum of ZXZTCs (Figure 1B). Based on the retention times and relative molecular masses of these metabolites, data from the literature (Luo et al., 2003; La et al., 2015; Pan, 2015; Wu et al., 2016; Zan et al., 2018), and comparisons with the standard substance, we identified five iridoid glycosides, five flavonoids, and one phenylethanol glycoside metabolites that entered the blood. The details are presented in Table 2. The metabolites detected included iridoid glycosides (shanzhiside, shanzhiside methyl ester, 8-O-acetyl shanzhiside methyl ester, 7-deoxyloganic acid, and notohamosin B), flavonoids (hyperoside, luteoloside, eugenyl-β-d-glucopyranoside, 7-methoxyapigenin, and apigenin-7-O-β-d-glucoside), and a phenylethanol glycoside (betonyoside A). The analytical features of the five metabolites of ZXZTCs detected in blood are listed below.

**TABLE 2:** MS data of ZXZTCs metabolites that were found in the blood.

Number	t_R_ (min)	Chemical formula	Molecular mass	ESI^−^ and its fragment ions	ESI^+^ and its fragment ions	Chemical name	Classification	References
1	4.33	C_17_H_26_O_11_	406	451	_	Shanzhiside methyl ester^a^	Iridoid glycoside	Pan (2015)
2	5.51	C_16_H_22_O_7_	326	325	327	Eugenyl-β-D-glucopyranoside	Flavonoid	La et al. (2015)
				651	349			
3	7.03	C_16_H_12_O_5_	284	283	_	7-Methoxyapigenin	Flavonoid	Wu et al. (2016)
4	8.47	C_16_H_24_O_9_	360	359	_	7-Deoxyloganic acid	Iridoid glycoside	Wu et al. (2016)
5	8.84	C_19_H_28_O_12_	448	493	_	8-O-acetyl shanzhiside methyl ester^a^	Iridoid glycoside	Pan, 2015; Zan et al. (2018)
6	16.40	C_21_H_20_O_12_	464	463	465	Hyperoside	Flavonoid	Wu et al. (2016)
7	16.53	C_16_H_24_O_11_	392	391	_	Shanzhiside	Iridoid glycoside	Wu et al. (2016)
8	17.49	C_21_H_20_O_11_	448	447	449	Luteoloside^a^	Flavonoid	Pan (2015)
9	18.00	C_21_H_20_O_10_	432	431	387	Apigenin-7-β-D-glucoside	Flavonoid	Pan (2015)
10	21.03	C_29_H_46_O_4_	458	457	481	Notohamosin B	Iridoid glycoside	Luo et al. (2003), La et al. (2015)
11	22.26	C_30_H_38_O_16_	654	653	_	Betonyoside A	Phenylethanol glycoside	Wu et al. (2016)

^a^ Indicates a comparison with the standard substance.

**FIGURE 6:**
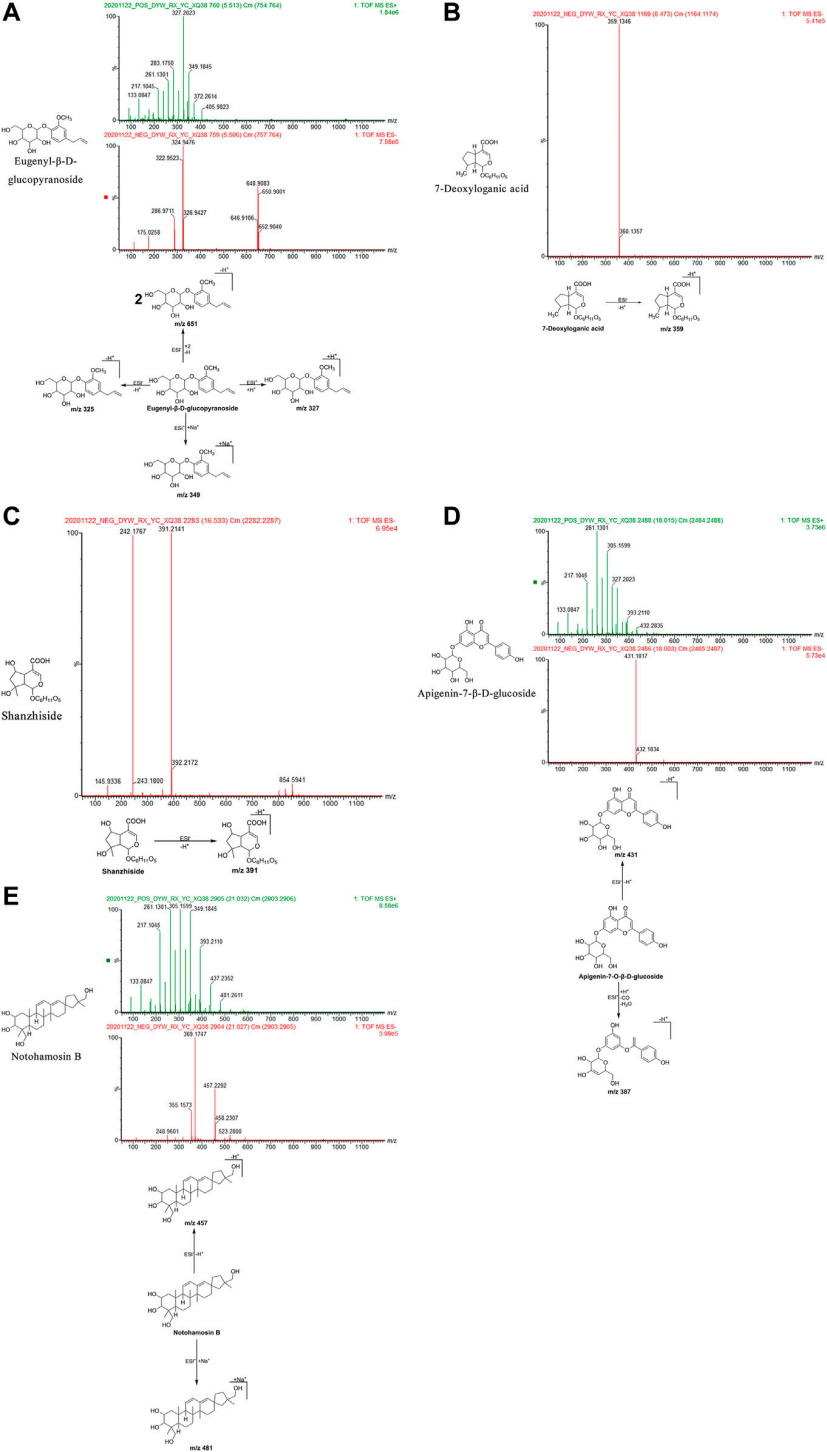
Structural formulas, mass spectra, and fragmentation processes of ZXZTCs metabolites that were found in the blood. **(A)** Eugenyl-β-D-glucopyranoside. **(B)** 7-Deoxyloganic acid. **(C)** Shanzhiside. **(D)** Apigenin-7-O-β-D-glucoside. **(E)** Notohamosin B.

Eugenyl-β-d-glucopyranoside: The retention time in the UPLC system was 5.51 min. In the ESI negative ion mode, a quasi-molecular ion peak at m/z 325 [M-H]^–^ and molecular ion peak at m/z 651 [2M-H]^–^ were detected near molecular ions 324, 322, 650, and 648. In the ESI positive ion mode, a quasi-molecular ion peak at m/z 327 [M + H]^+^appeared, along with a molecular ion peak at m/z 349 [M + Na]^+^. The molecular mass of the metabolite was confirmed as 326. Based on the present findings in combination with earlier literature (La et al., 2015), the metabolite was inferred as eugenyl-β-d-glucopyranoside with a molecular formula of C_16_H_22_O_7_. The mass spectrum, structural formula, and fragmentation process are shown in Figure 6A.

7-Deoxyloganic acid: The retention time in the UPLC system was 8.47 min. In the ESI negative ion mode, a quasi-molecular ion peak at m/z 359 [M-H]^–^ was obtained. The molecular mass of the metabolite was confirmed as 360. Based on the present findings in combination with earlier literature (Wu et al., 2016), the metabolite was inferred as 7-deoxyloganic acid with a molecular formula of C_16_H_24_O_9_. The mass spectrum, structural formula, and fragmentation process are shown in Figure 6B.

Shanzhiside: The retention time in the UPLC system was 16.53 min. In the ESI negative ion mode, a quasi-molecular ion peak at m/z 391 [M-H]^–^ was obtained. The molecular mass of the metabolite was confirmed as 392. Based on the present findings in combination with earlier literature (Wu et al., 2016), the metabolite was inferred as shanzhiside with a molecular formula of C_16_H_24_O_11_. The mass spectrum, structural formula, and fragmentation process are shown in Figure 6C.

Apigenin-7-β-d-glucoside: The retention time in the UPLC system was 18.00 min. In the ESI negative ion mode, a quasi-molecular ion peak m/z 431 [M-H]^–^ was obtained. In the ESI positive ion mode, fragment ions of a molecular ion peak at m/z 387 [M + H-CO-H_2_O]^+^were obtained. The molecular mass of the metabolite was confirmed as 432. Based on the present findings in combination with earlier literature (Pan, 2015), the metabolite was inferred as apigenin-7-O-β-d-glucoside with a molecular formula of C_21_H_20_O_10_. The mass spectrum, structural formula, and fragmentation process are shown in Figure 6D.

Notohamosin B: The retention time of the UPLC system was 21.03 min. In the ESI negative ion mode, a quasi-molecular ion peak at m/z 457 [M-H]^–^ was obtained. In the ESI positive ion mode, a molecular ion peak at m/z 481 [M + Na]^+^was detected. The molecular mass of the metabolite was confirmed as 458. Based on the present findings in combination with earlier literature (La et al., 2015), the metabolite was inferred as notohamosin B with a molecular formula of C_29_H_46_O_4_. The mass spectrum, structural formula (Luo et al., 2003), and fragmentation process are shown in Figure 6E.

### 3.3 HPLC determination of the six metabolites of ZXZTCs

The batch numbers of the six batches of ZXZTCs tested in this experiment were 180902, 200801, 200802, 190101, 201101, and 190201.

#### 3.3.1 Methodological investigation

We prepared 0.625, 1.25, 2.5, and 5 mL of mixed standard solution for HPLC as described in the ‘Preparation of the test and standard solutions’ section. Solutions were placed in a 10 mL volumetric flask and diluted to the required volume with methanol to obtain a series of concentrations of mixed standard solution. Taking the mixed standard solution of each series of concentrations and the mixed standard solution for HPLC as described in the ‘Preparation of test and standard solutions’ section, 10 μL aliquots were injected into the liquid chromatography system, and peak areas were measured. The concentration was taken as the abscissa and the peak area was taken as the ordinate to obtain the standard curve equation for each component (Kongkiatpaiboon et al., 2017). The peak area of each standard substance was linearly related to the concentration (Supplementary Table S1). A 10 μL aliquot of the mixed standard solution was precisely prepared for HPLC, as described in the ‘Preparation of test and standard solutions’ section, and was injected six times continuously within 1 day. The peak area of each component was recorded (Alfredo et al., 2019). The results showed that the relative standard deviation of each standard substance was <3%, indicating good intraday precision of the instrument, as shown in Supplementary Table S1. Approximately 0.5 g of ZXZTCs powder, a total of 6 parts, were weighed accurately, and 10 μL of test solution of each ZXZTCs was accurately sucked according to the preparation method described in the ‘Preparation of test and standard solutions’ section for HPLC. Solutions were injected into the liquid chromatography system, the peak areas were measured, and RSD values were calculated. Notably, the RSD of each standard substance was <3%, indicating good repeatability of the method, as shown in Supplementary Table S1. Approximately 0.5 g of ZXZTCs powder, 10 μL of test solution of ZXZTCs, was accurately sucked according to the preparation method described in the ‘Preparation of test and standard solutions’ section for HPLC. Solutions were injected into the liquid chromatography system at 0, 2, 4, 8, 12, and 24 h, the peak areas were recorded, and RSD values were calculated. The RSD of each standard substance was <3%, indicating that the test solution was stable within the 24 h experimental period, as shown in Supplementary Table S1.

#### 3.3.2 Content determination

Approximately 0.5 g of powder (capsule content) from each of six batches (batch numbers 180902, 200801, 200802, 190101, 201101, and 190201) was accurately weighed. Three parallel experiments were performed for each batch. Samples were accurately weighed and prepared according to the method for HPLC described in the ‘Preparation of test and standard solutions’ section. A precise 10 μL aliquot of the test solution was injected and the chromatography peak area of each component was recorded. The contents were measured, and average values were calculated (Table 3). The metabolite contents from high to low levels were as follows: forsythin B, 8-O-acetyl shanzhiside methyl ester, luteoloside, verbascoside, shanzhiside methyl ester, and chlorogenic acid. The content chromatograms for each batch are shown in Figure 1F.

**TABLE 3:** The contents of six metabolites in ZXZTCs (*n* = 6).

Batch number		Component content (mg·g^-1^)					
		Shanzhiside methyl ester	Chlorogenic acid	8-O-acetyl shanzhiside methyl ester	Forsythin B	Luteoloside	Verbascoside
180902	1	1.4145	1.1419	1.9334	4.2727	1.9775	1.6338
	2	1.4312	1.1495	1.9427	4.3239	1.9729	1.6003
	3	1.4061	1.1375	1.9164	4.3998	1.9721	1.6262
	Average value	1.42	1.14	1.93	4.33	1.97	1.62
	Standard deviation	0.01	0.01	0.01	0.06	0.00	0.02
200801	1	1.9780	1.2776	3.7338	5.8037	2.237	1.8222
	2	1.9710	1.2481	3.6769	5.8932	2.2281	1.8409
	3	2.1447	1.3753	3.8444	6.2927	2.4142	1.8356
	Average value	2.03	1.30	3.75	6.00	2.29	1.83
	Standard deviation	0.10	0.07	0.09	0.26	0.10	0.01
200802	1	2.0928	1.2888	3.9128	6.1457	2.3929	1.8624
	2	2.0947	1.3204	3.8878	5.5670	2.3934	1.8508
	3	2.1105	1.3040	3.9484	5.6916	2.4230	1.8678
	Average value	2.10	1.30	3.92	5.80	2.40	1.86
	Standard deviation	0.01	0.02	0.03	0.30	0.02	0.01
190101	1	1.1080	0.6532	1.1882	2.4334	1.3781	0.9648
	2	1.0853	0.6447	1.1630	2.4623	1.3447	0.8908
	3	1.1169	0.6569	1.1592	2.5311	1.3571	0.9295
	Average value	1.10	0.65	1.17	2.48	1.36	0.93
	Standard deviation	0.02	0.01	0.02	0.05	0.02	0.04
201101	1	3.2887	3.4016	7.7340	9.1611	3.3344	2.9664
	2	3.2431	3.3569	8.0384	8.3309	3.3209	2.9446
	3	3.2076	3.4205	8.0424	8.6169	3.4245	3.0934
	Average value	3.25	3.39	7.94	8.70	3.36	3.00
	Standard deviation	0.04	0.03	0.18	0.42	0.06	0.08
190201	1	0.9532	0.8942	1.6250	5.4941	2.4044	2.1688
	2	0.9455	0.4572	1.6215	5.5556	2.4316	2.2063
	3	0.95507	0.9494	1.6572	5.3658	2.4106	2.1374
	Average value	0.95	0.77	1.63	5.47	2.42	2.17
	Standard deviation	0.01	0.27	0.02	0.10	0.01	0.03
**Total average value**		1.81	1.43	3.39	5.46	2.30	1.90
**Total relative standard deviation**		0.80	0.95	2.35	1.94	0.62	0.64

### 3.4 Hemostatic effect of ZXZTCs

#### 3.4.1 Effects of ZXZTCs on the bleeding and coagulation times of severed tails in mice

Details are presented in Table 4. Compared with the blank control group, the tail bleeding times of mice in each drug treatment group were very significantly shortened (*p* < 0.01), whereas no significant changes were observed in coagulation time (*p* > 0.05). Compared with the carbazochrome control group, the bleeding and coagulation times of mice in each drug treatment group showed no significant changes (*p* > 0.05). Our findings indicate that ZXZTCs stop bleeding by shortening the bleeding time. The action strength (from strong to weak) of the preparation was in the following order: medium dose, high dose, and low dose.

**TABLE 4:** Changes in bleeding and coagulation times after tail amputation in mice, blood agglutination parameters, and plasma recalcification times in rats (*n* = 10, $\bar{x}\pm s$ ).

Group	Dose	Bleeding and coagulation times of mice with severed tails			Blood agglutination parameters and plasma recalcification times in rats			
		Bleeding time (s)	Coagulation time (s)	Prothrombin time (s)		Thrombin time (s)	Fibrinogen concentration (mg/dL)	Plasma recalcification time (s)
Blank control group	—	118.00 ± 47.56	56.00 ± 15.78	9.19 ± 0.38		17.95 ± 1.24	154.20 ± 13.49	100.00 ± 18.86
Carbazochrome control group	0.0025 g/kg/d	36.00 ± 26.33**	42.00 ± 14.76	—		—	—	—
Yunnan Baiyao capsules control group	0.1667 g/kg/d	—	—	9.29 ± 0.46		19.61 ± 1.32**	163.00 ± 12.15	60.00 ± 16.33**
Low-dose ZXZTCs group	Mice: 0.2625 g/kg/d	46.00 ± 21.19**	64.00 ± 15.78	9.34 ± 0.45		19.29 ± 1.01*	158.20 ± 117.84	82.00 ± 17.51** ^##^
	Rat: 0.1313 g/kg/d							
Medium-dose ZXZTCs group	Mice: 0.5250 g/kg/d	34.00 ± 18.97**	56.00 ± 18.38	9.57 ± 0.41		18.14 ± 1.26^##^	156.80 ± 17.72	70.00 ± 14.14**
	Rat: 0.2625 g/kg/d							
High-dose ZXZTCs group	Mice: 1.0500 g/kg/d	38.00 ± 33.26**	44.00 ± 12.65	9.45 ± 0.65		19.64 ± 0.98**	151.50 ± 10.49	74.00 ± 9.66** ^#^
	Rat: 0.5250 g/kg/d							

Note: bleeding and coagulation times of mice with severed tails: compared with the blank control group, ^*^ *p* < 0.05, ^**^ *p* < 0.01; compared with the carbazochrome control group, ^*#*^ *p* < 0.05, ^*##*^ *p* < 0.01.

Blood agglutination parameters and plasma recalcification times of rats: compared with the blank control group, ^*^ *p* < 0.05,^**^ *p* < 0.01; Compared with the Yunnan Baiyao capsules control group, ^*#*^ *p* < 0.05, ^*##*^ *p* < 0.01.

#### 3.4.2 Effects of ZXZTCs on blood agglutination parameters and plasma recalcification time in rats

Details are presented in Table 4. Compared with the blank control group, the thrombin time of rats in the low-dose group of ZXZTCs was significantly prolonged (*p* < 0.05); the thrombin times of animals in the Yunnan Baiyao capsules control group and high-dose ZXZTCs group were prolonged to a more significant extent (*p* < 0.01); the plasma recalcification time of rats in each drug group was very markedly decreased (*p* < 0.01). Compared with the Yunnan Baiyao capsules control group, the plasma recalcification time of rats in the low-dose ZXZTCs group was very significantly prolonged (*p* < 0.01); the thrombin time of rats in the medium-dose ZXZTCs group was decreased to a highly significant extent (*p* < 0.01); the plasma recalcification time of rats in the high-dose ZXZTCs group was significantly prolonged (*p* < 0.05). Based on the results, we speculate that ZXZTCs has a two-way regulatory effect, promoting either coagulation or anti-coagulation. On the one hand, all three doses of ZXZTCs shortened plasma recalcification time in rats, which is indicative of a potential procoagulant effect. On the other hand, low and high doses of ZXZTCs prolonged the thrombin time of rats, signifying a potential anticoagulant effect.

#### 3.4.3 Effects of ZXZTCs on platelets and aggregation rates in rats

Details are presented in Table 5. Compared with the blank control group, the numbers of leukocytes, lymphocytes, and monocytes in the low-dose ZXZTCs group were significantly increased (*p* < 0.05); the platelet aggregation rates of rats in low- and medium-dose ZXZTCs groups was significantly decreased (*p* < 0.05). Our results show that ZXZTCs reduce platelet aggregation but have no significant effect on the number of platelets. The action strength of the preparation (from strong to weak) was in the following order: low dose, medium dose, and high dose.

**TABLE 5:** Changes of platelets and their aggregation rates in rats (*n*=10, $\bar{x}\pm s$ ).

Group	Dose	Platelet and their aggregation rate of rats								
		Platelet count (PLT) 10E9/L	Mean platelet volume (MPV) fL	Platelet distribution width (PDW)%	Platelet hematocrit (PCT) %	Red blood cells count (RBC) 10E12/L	Hemoglobin (HGB) g/L	Hematocrit (HCT)%	Mean red blood cell volume (MCV) fL	Mean corpuscular hemoglobin content (MCH) pg
Blank control group	——	1397.80±205.89	5.54±0.31	16.06±0.25	0.66±0.03	7.02±1.01	145.10±13.58	42.38±4.13	60.86±3.68	20.76±1.25
Yunnan Baiyao capsules control group	0.1667g/kg/d	1604.44±265.39	5.69±0.24	16.04±0.16	——	7.10±0.69	146.78±12.69	42.67±4.03	60.81±2.08	20.66±0.88
Low-dose ZXZTCs group	0.1313g/kg/d	1501.00±242.47	5.59±0.32	16.01±0.26	0.66±0.06	7.55±0.52	155.40±8.50	45.71±2.76	60.72±2.12	20.55±0.72
Medium-dose ZXZTCs group	0.2625g/kg/d	1390.22±171.97	5.67±0.30	16.07±0.21	0.64±0.08	7.33±0.59	154.44±9.04	45.33±2.63	62.09±2.86	21.07±0.87
High-dose ZXZTCs group	0.5250g/kg/d	1403.78±91.92	5.61±0.39	16.06±0.14	0.70±0.00	7.36±0.54	154.78±8.27	45.23±2.23	61.64±1.83	21.01±0.51

Group	Platelet and their aggregation rate of rats									
	Mean corpuscular hemoglobin concentration (MCHC) g/L	Coefficient of variation of erythrocyte distribution width (RDW) %	White blood cell count (WBC) 10E9/L	Lymphocyte count (Lymph#) 10E9/L	Monocytes count (Mon#) 10E9/L	Neutrophils count (Gran#) 10E9/L	Percentage of lymphocytes (lymph%)	Percentage of monocytes (mon%)	Percentage of neutrophils (gran%)	Platelet aggregation rate (PAG)%
Blank control group	342.10±6.38	11.96±1.41	5.48±3.20	3.89±2.27	0.14±0.07	1.55±1.01	71.88±7.11	2.76±0.60	25.36±6.93	32±7.3
Yunnan Baiyao capsules control group	340.11±6.92	12.00±2.07	6.26±3.40	4.74±2.64	0.13±0.09	1.38±0.81	74.42±6.77	2.41±0.67	23.17±6.24	29.2±11
Low-dose ZXZTCs group	339.60±3.24	11.74±0.93	8.54±1.84*	6.20±1.56*	0.22±0.08*	2.12±0.90	72.60±10.22	2.62±0.61	24.78±9.73	24.9±3.8*
Medium-dose ZXZTCs group	340.22±6.26	11.57±0.92	5.53±3.07	4.12±2.34	0.12±0.10	1.29±0.66	71.99±6.25	2.82±0.74	25.19±5.56	25.4±5.2*
High-dose ZXZTCs group	341.56±5.15	11.92±0.86	8.70±4.37	6.69±3.52	0.21±0.31	1.80±0.88	75.76±6.33	2.50±0.37	21.74±6.22	29.8±7.1

Note: compared with the blank control group, ^*^ *p* < 0.05, ^**^ *p* < 0.01.

#### 3.4.4 Effects of ZXZTCs on the model of thrombocytopenia induced by cytarabine in mice

Details are presented in Table 6. Compared with the blank control group, the number of platelets in the model control group was significantly increased (*p* < 0.05), concomitant with a highly significant increase in hematocrit levels (*p* < 0.01). Compared with the model control group, the number of erythrocytes and hematocrit levels in the prednisone acetate control group were significantly increased (*p* < 0.05); the average erythrocyte hemoglobin content was significantly decreased (*p* < 0.05), the number of monocytes was significantly increased (*p* < 0.05), and the percentage of monocytes was highly significantly increased (*p* < 0.01) in the low-dose ZXZTCs group; the number of monocytes in the medium-dose ZXZTCs group was significantly increased (*p* < 0.05), while average erythrocyte volume and erythrocyte hemoglobin content were highly significantly decreased (*p* < 0.01); the number of leukocytes and percentage of monocytes in the high-dose ZXZTCs group were significantly increased (*p* < 0.05), along with a highly significant increase in the number of monocytes (*p* < 0.01); the organ coefficients of spleen and thymus in each drug group showed no significant changes (*p* > 0.05). These findings showed that there were no noteworthy effects of ZXZTCs on the cytarabine-induced thrombocytopenia model in mice.

**TABLE 6:** Changes in various indices of the thrombocytopenia model induced by cytarabine in mice (n=10, $\bar{x}\pm s$ ).

Group	Dose	Mice thrombocytopenia model induced by cytarabine									
		Platelet count (PLT) 10E9/L	Mean platelet volume (MPV) fL	Platelet distribution width (PDW)%	Platelet hematocrit (PCT) %	Red blood cells count (RBC) 10E12/L	Hemoglobin (HGB) g/L	Hematocrit (HCT)%	Mean red blood cell volume (MCV) fL	Mean corpuscular hemoglobin content (MCH) pg	Mean corpuscular hemoglobin concentration (MCHC) g/L
Blank control group	——	1314.50±535.35	5.94±0.59	16.62±0.58	0.49±0.16	9.46±2.21	153.90±36.19	52.46±13.53	55.20±2.29	16.30±1.82	296.30±33.02
Model control group	——	459.80±120.35^##^	5.86±0.34	16.97±0.42	0.27±0.06^##^	7.72±0.56^#^	134.50±9.59	42.66±2.70^#^	55.37±2.09	17.37±0.49	314.50±5.60
Prednisone acetate tablets control group	0.0100 g/kg/d	489.90±344.19	6.12±0.56	17.15±0.55	0.24±0.12	8.42±0.59^*^	142.50±7.49	45.21±2.35^*^	53.83±1.89	16.91±0.86	315.00±14.59
Low-dose ZXZTCs group	0.2625g/kg/d	532.50±170.70	5.57±0.30	16.69±0.52	0.29±0.09	8.26±0.66	138.10±7.99	43.75±2.22	53.15±2.57^*^	16.72±0.71^*^	315.10±5.84
Medium-dose ZXZTCs group	0.5250g/kg/d	501.10±200.34	5.92±0.29	16.86±0.33	0.29±0.11	8.38±1.32	138.00±23.45	43.79±7.11	52.31±1.69^**^	16.40±0.51^**^	314.50±4.67
High-dose ZXZTCs group	1.0500g/kg/d	599.20±433.96	6.00±0.56	17.04±0.50	0.28±0.08	7.61±0.34	129.90±7.50	41.40±2.61	54.50±2.63	17.01±0.80	313.40±4.74

Group	Mice thrombocytopenia model induced by cytarabine									
	Coefficient of variation of erythrocyte distribution width (RDW) %	White blood cell count (WBC) 10E9/L	Lymphocyte count (lymph#) 10E9/L	Monocytes count (mon#) 10E9/L	Neutrophils count (gran#) 10E9/L	Percentage of lymphocytes (lymph%)	Percentage of monocytes (mon%)	Percentage of neutrophils (gran%)	Spleen coefficient	Thymus coefficient
Blank control group	15.90±1.22	2.09±0.99	1.47±1.08	0.08±0.09	0.54±0.39	63.71±22.83	5.18±3.27	31.11±20.20	0.00570±0.00130	0.00357±0.00071
Model control group	15.45±2.08	0.96±0.31^##^	0.80±0.24	0.00±0.00^#^	0.21±0.13^#^	75.13±9.31	4.17±1.49	20.70±7.93	0.00528±0.00177	0.00149±0.00036^##^
Prednisone acetate tablets control group	15.32±1.43	0.84±0.48	0.84±0.34	0.03±0.05	0.23±0.10	72.21±17.67	4.51±3.59	23.27±14.15	0.00444±0.00091	0.00108±0.00125
Low-dose ZXZTCs group	15.67±2.33	1.00±0.19	0.71±0.14	0.05±0.05^*^	0.24±0.10	70.36±6.57^^^	6.17±1.39^**^^	23.47±5.47	0.00511±0.00152	0.00119±0.00038
Medium-dose ZXZTCs group	16.27±1.97	0.88±0.30	0.70±0.21	0.04±0.05^*^	0.18±0.10	72.11±8.92	5.63±1.86	22.26±7.19	0.00530±0.00190	0.00118±0.00044
High-dose ZXZTCs group	15.77±1.44	1.46±0.52^*^	1.08±0.37	0.06±0.05^**^	0.32±0.13	73.28±5.12	5.63±1.20^*^	21.09±4.13	0.00625±0.00163	0.00120±0.00035

Note: compared with the blank control group, ^*#*^ *P*<0.05,^*##*^ *P*<0.01; compared with the model control group, ^*^ *P*<0.05,^**^ *P*<0.01.

#### 3.4.5 Effects of ZXZTCs on tail thrombosis induced by carrageenan in mice

Details are presented in Table 7. Compared with the model control group, at 24, 48, and 72 h, the tail thrombosis rates of the aspirin enteric-coated tablets control group and medium-dose ZXZTCs group were significantly decreased (*p* < 0.05). Compared with the aspirin enteric-coated tablets control group, the tail thrombosis rates of mice in low- and high-dose ZXZTCs groups were significantly higher at 24, 48, and 72 h (*p* < 0.05); at all three time points, there were no significant differences in the tail thrombosis rates of mice in the medium-dose ZXZTCs group (*p* > 0.05), suggesting similar strengths of action of the two groups (the medium-dose group of ZXZTCs and the aspirin enteric-coated tablet control group). This finding indicates that ZXZTCs can reduce tail thrombosis in mice. The effect strength of the medium dose was the greatest, equivalent to that of aspirin enteric-coated tablets, followed by low and high doses of ZXZTCs.

**TABLE 7:** Changes of various indices of the tail thrombosis model induced by carrageenan in the mice experiment and the *in vitro* Chandler thrombosis model of rats experiment (*n* = 10, $\bar{x}\pm s$ ).

Group	Dose	Tail thrombosis rate (tail thrombosis model induced by carrageenan in mice)			Thrombus change (*in vitro* chandler thrombus model of rats)		
		24 h (%)	48 h (%)	72 h (%)	Thrombus length (mm)	Thrombus dry weight (mg)	Thrombus wet weight (mg)
Model control group	—	35.13 ± 10.77	35.94 ± 12.72	34.70 ± 12.87	19.02 ± 2.19	22.79 ± 4.34	90.41 ± 15.57
Aspirin enteric-coated tablets control group	Mice: 0.05 g/kg/d	23.26 ± 11.63*	22.34 ± 10.99*	22.09 ± 10.79^*^	16.86 ± 1.48^*^	18.76 ± 2.80^*^	70.08 ± 10.19^**^
	Rat: 0.025 g/kg/d						
Low-dose ZXZTCs group	Mice: 0.2625 g/kg/d	32.41 ± 7.57^#^	30.36 ± 5.85^#^	29.34 ± 4.84^#^	19.08 ± 2.41^#^	20.97 ± 4.15	78.51 ± 14.94
	Rat: 0.1313 g/kg/d						
Medium-dose ZXZTCs group	Mice: 0.5250 g/kg/d	25.32 ± 6.45^*^	24.01 ± 6.60^*^	24.08 ± 6.74^*^	18.99 ± 2.67^#^	22.27 ± 3.36^#^	91.87 ± 17.79^#^
	Rat: 0.2625 g/kg/d						
High-dose ZXZTCs group	Mice: 1.0500 g/kg/d	37.93 ± 10.59^#^	35.05 ± 10.58^#^	33.28 ± 9.26^#^	17.04 ± 1.97^*^	18.54 ± 4.09^*^	75.37 ± 14.78^*^
	Rat: 0.5250 g/kg/d						

Note: mice tail thrombosis induced by carrageenan: compared with the model control group, *p < 0.05, **p < 0.01; Compared with aspirin enteric-coated tablets control group, ^#^ *p* < 0.05, ^##^ *p* < 0.01.

*In vitro* rat Chandler thrombus model: Compared with the model control group, *p < 0.05, **p < 0.01; compared with the aspirin enteric-coated tablets control group, ^#^ *p* < 0.05, ^##^ *p* < 0.01.

#### 3.4.6 Effects of ZXZTCs on the chandler thrombus model *in vitro* of rats

Details are presented in Table 7. Compared with the blank control group, the length and dry weight of thrombi in the aspirin enteric-coated tablets control group were significantly decreased (*p* < 0.05), along with a highly significant decrease in the wet weight of thrombus (*p* < 0.01); thrombus length and thrombus dry and wet weights in the high-dose ZXZTCs group were significantly decreased (*p* < 0.05). Compared with the aspirin enteric-coated tablets control group, thrombus length of rats in the low-dose ZXZTCs group was significantly higher (*p* < 0.05); moreover, the length, dry weight, and wet weight of thrombus of rats in the medium-dose ZXZTCs group were significantly increased (*p* < 0.05); no marked differences were observed in thrombus length, thrombus dry weight, and thrombus wet weight in the high-dose ZXZTCs group of (*p* > 0.05), suggesting similar action strengths of the two treatments (the high-dose ZXZTCs group and the aspirin enteric-coated tablets control group). Our results indicate that ZXZTCs can effectively reduce thrombosis in rats. The effect strength of the high dose was the greatest, equivalent to that of aspirin enteric-coated tablets, followed by low and medium doses.

### 3.5 Analgesic effect of ZXZTCs

#### 3.5.1 Effects of ZXZTCs on writhing response induced by acetic acid in mice

Details are presented in Table 8. Compared with the model control group, the latency of writhing was significantly prolonged (*p* < 0.05), and writhing was significantly reduced in the low-dose ZXZTCs group (*p* < 0.05); prolongation of the latency of writhing was highly significant (*p* < 0.01) in the aspirin enteric-coated tablets control group and medium- and high-dose ZXZTCs groups, along with very markedly reduced writhing (*p* < 0.01). Compared with the aspirin enteric-coated tablets control group, the latency of writhing in mice in the low-dose ZXZTCs group was very significantly shortened (*p* < 0.01); writhing of mice in the low-, medium-, and high-dose ZXZTCs groups was highly significantly increased (*p* < 0.01). Overall, ZXZTCs could prolong the latency of writhing, reduce writhing, and produce analgesic effects in mice. The effect strength of the high dose was the greatest, followed by medium and low doses. The effect strength of the medium and high doses of ZXZTCs in terms of prolonging latency of writhing in mice was equivalent to that of aspirin enteric-coated tablets.

**TABLE 8:** Changes of various indices in animals in analgesic experiments (*n* = 10, $\bar{x}\pm s$ ).

Group	Dose	Writhing response induced by acetic acid in mice			Foot pain induced by a hot plate in mice				
		Latency of writhing (S)	Writhing (times)	Analgesia percentage (%)	Pain threshold before administration (S)	Pain threshold (s, first row values for each group) Percentage of pain threshold increase (%, second row values for each group)			
						30 min	60 min	90 min	120 min
Model control group	——	199.6±48.2	29.4±5.5	——	19.5±3.76	14.9±6.23	19.4±7.78	17.3±9.57	17.7±9.74
						-22.7±32.62	-0.8±33.57	-10.53±46	-6.74±54.64
Aspirin enteric-Coated tablets control group	0.05g/kg/d	321.4±52.8**	9.2±2.6**	68.71	20.7±3.11	29.3±11.83^#^**	24.9±9.52	23.1±15.45	27.6±13.52
						46.38±62.36**	23.93±56.73	14.36±73.88	33.45±66.76
Rotundine tablets control group	0.030g/kg/d	——	——	——	——	——	——	——	——
Ibuprofen capsules control group	0.1333g/kg/d	——	——	——	——	——	——	——	——
Low-dose ZXZTCs group	Mice: 0.05g/kg/d	254.3±49.6*^##^	23.7±6.2*^##^	19.39	21.8±4.78	21.8±9.43	19±7.67	26.6±18.28	25.6±18.83
	Rat: 0.025g/kg/d					-2.58±30.48^*$*^	-12.67±30.73	19.21±66.88	11.79±60.86
Medium-dose ZXZTCs group	Mice: 0.2625g/kg/d	280.3±46.1**	18.7±5.7**^##^	36.39	19.1±5.41	16.5±8.55	19.3±12.94	28.2±20.71	36±23.37^#^*
	Rat: 0.1313g/kg/d					-10.62±48.63^*$*^	9.3±86.7	62.01±148.87	96.84±146.36
High dose ZXZTCs group	Mice: 0.5250g/kg/d	298.3±49.9**	14.0±5.0**^##^	51.36	21.5±4.76	22.3±8.71*	20±17	31.6±21.18	36.3±20.38^#^*
	Rat: 0.2625g/kg/d					13.88±68.93	-3.43±85.75	61.93±141.26	80.98±132.83

Group	Foot tenderness induced by mechanical stimuli in rats					Tail flick response induced by photothermal stimulation in rats					Dysmenorrhea model induced by oxytocin in mice		
	Pain threshold before administration (S)	Pain threshold (s, first row values for each group) Percentage of pain threshold increase (%, second row values for each group)				Pain threshold before administration (S)	Pain threshold (s, first row values for each group) Percentage of pain threshold increase (%, second row values for each group)				Latency of writhing (S)	Writhing (times)	Analgesia percentage (%)
		30 min	60 min	90 min	120 min		30 min	60 min	90 min	120 min			
Model control group	240.70±32.06	233.20±60.03	245.95±21.4	244.12±34.90	266.38±39.67	5.13±1.4	4.54±1.68	4.58±0.81	5.94±1.52	4.42±1.06	426.1±47.51	13.9±2.33	——
		0.010±32.23	4.03±18.31	3.36±23.00	12.86±25.34		-4.69±40.75	-4.46±30.10	25.28±48.04	-6.94±34			
Aspirin enteric-Coated tablets control group	——	——	——	——	——	——	——	——	——	——	——	——	——
Rotundine tablets control group	240.53±36.85	283.34±37.83^#^*	302.62±53.5^##^**	291.12±59.64^#^*	289.06±57.61^#^	4.03±1.22	11.41±3.34^##^**	13.92±2.48^##^**	14.57±4.49^##^**	13.53±3.41^##^**	——	——	——
		19.56±19.77	28.74±30.78*	23.28±29.50	21.34±21.52		216.23±168.15**	279.63±165.38**	297.67±194.72**	273.50±177.77**			
Ibuprofen capsules control group	——	——	——	——	——	——	——	——	——	——	1283.8±94.84**	3.4±1.43**	75.54
Low-dose ZXZTCs group	230.38±33.94	246.07±41.14	276.19±69.41	259.84±37.82	266.26±64.36	4.45±1.24	5.02±1.18	5.35±1.29	5.71±1.51	5.26±2.14	676.9±119.30**^##^	11.4±3.34^##^	17.99
		7.55±17.59	20.26±26.69	13.91±17.55	16.39±29.60		19.46±36.44^*$$*^	30.79±51.78^*$$*^	35.33±45.61^*$$*^	35.71±78.98^*$$*^			
Medium-dose ZXZTCs group	225.45±37.58	246.70±31.45	291.33±52.56^##^*	280.05±40.72^##^*	249.46±49.57	5.17±1.47	5.47±2.10	6.16±1.78*	6.29±1.45	5.40±1.64	809.6±94.26**^##^	8.4±2.67**^##^	39.57
		5.59±15.83	26.16±31.59	21.29±28.09	8.22±28.43		15.91±56.39^*$$*^	30.05±53.68^*$$*^	29.52±42.08^*$$*^	14.75±52.49^*$$*^			
High dose ZXZTCs group	230.19±44.95	274.06±48.10^#^	320.00±93.00^#^*	264.95±46.15	240.98±78.36	4.4±1.36	6.33±2.06^#^*	7.00±2.00^##^**	7.48±2.32^##^	5.82±1.93	1098.5±140.69**^##^	5.0±2.00**^#^	61.87
		24.79±42.67	46.52±59.11	19.05±28.99	10.10±44.04		60.60±70.75*^$^	73.34±61.32**^$$^	81.6±74.14^*$$*^	43.44±57.13*^$$^			

Note: writhing response in mice induced by acetic acid: compared with the model control group, ^*^ *p* < 0.05, ^**^ *p* < 0.01; compared with the aspirin enteric-coated tablets control group, ^#^ *p* < 0.05, ^##^ *p* < 0.01.

Foot pain in mice induced by a hot plate: compared with pain threshold before administration, ^#^ *p* < 0.05, ^##^ *p* < 0.01; compared with the model control group, ^*^ *p* < 0.05, ^**^ *p* < 0.01; compared with the aspirin enteric-coated tablets control group, ^$^ *p* < 0.05, ^$$^ *p* < 0.01.

Foot tenderness in rats induced by mechanical stimuli: compared with pain threshold before administration, ^#^ *p* < 0.05, ^##^ *p* < 0.01; compared with the model control group, *p < 0.05, ** *p* < 0.01; compared with the rotundine tablets control group, ^$^ *p* < 0.05, ^$$^ *p* < 0.01.

Tail flick response in rats induced by photothermal stimulation: compared with pain threshold before administration, ^#^ *p* <0.05, ^##^ *p* < 0.01; compared with the model control group, ^*^ *p* < 0.05, ^**^ *p* < 0.01; compared with the rotundine tablets control group, ^$^ *p* < 0.05, ^$$^ *p* < 0.01.

Dysmenorrhea model in mice induced by oxytocin: compared with the model control group, ^*^ *p* < 0.05, ^**^ *p* < 0.01; compared with the ibuprofen capsules control group, ^#^ *p* < 0.05, ^##^ *p* < 0.01.

#### 3.5.2 Effects of ZXZTCs on foot pain induced by a hot plate in mice

Details are presented in Table 8. The pain threshold of aspirin enteric-coated tablets control group was significantly increased 30 min (*p* < 0.05) after drug administration; the pain thresholds of mice in the medium- and high-dose ZXZTCs groups were significantly increased at 120 min (*p* < 0.05).

Relative to the model control group, the increase in pain threshold of the aspirin enteric-coated tablets control group was highly significant at 30 min (*p* < 0.01), and the percentage of pain threshold was highly significantly increased at 30 min (*p* < 0.01); the pain threshold of mice in the medium-dose ZXZTCs group was significantly increased at 120 min (*p* < 0.05) and was significantly increased at 30 and 120 min (*p* < 0.05) in the high-dose ZXZTCs group. Compared with the control aspirin enteric-coated tablets group, the percentage of pain threshold increase in the low- and medium-dose ZXZTCs groups was significantly lower at 30 min (*p* < 0.05); moreover, no significant changes were observed in the percentage of pain threshold increase at other time points and at all time points for the other groups (*p* > 0.05).

Our results showed that ZXZTCs can increase the threshold of foot pain in mice caused by a hot plate and subsequently achieve the effect of analgesia. The effect strength of the high dose was the greatest, followed by medium and low doses. The effect strength of high-dose ZXZTCs in terms of percentage increase in pain threshold was equivalent to that of aspirin enteric-coated tablets.

#### 3.5.3 Effects of ZXZTCs on foot tenderness induced by mechanical stimulation in rats

Details are presented in Table 8. The pain threshold of rats in the rotundine tablets control group was significantly increased 30 and 90–120 min after drug administration (*p* < 0.05) and highly significantly increased 60 min (*p* < 0.01) after drug administration; the increase in the pain threshold of rats in the medium-dose ZXZTCs group was highly significant during the 60–90 min period (*p* < 0.01) and the pain threshold of rats in the high-dose ZXZTCs group was significantly increased during the 30–60 min period (*p* < 0.05).

After drug administration, compared with the model control group, the pain threshold of the rotundine tablets control group was significantly greater at 30 and 90 min (*p* < 0.05) and highly significantly greater at 60 min (*p* < 0.01), along with the percentage of pain threshold increase at 60 min (*p* < 0.05); the pain threshold of rats in the medium-dose ZXZTCs group was significantly increased during the 60–90 min period (*p* < 0.05) and the pain threshold of rats in the high-dose ZXZTCs group was significantly increased at 60 min (*p* < 0.05). Compared with the rotundine tablets control group, the percentage of pain threshold increase in rats in low-, medium-, and high-dose ZXZTCs groups was not markedly different over 30–120 min (*p* > 0.05), suggesting that the action strength of all three doses of ZXZTCs was equivalent to that of the rotundine tablets.

Our results showed that ZXZTCs can increase the pain threshold of foot tenderness induced by mechanical stimulation and subsequently achieve the effect of analgesia. The effect strength of the high dose was the greatest, followed by medium and low doses. Moreover, the effect strength of the ZXZTCs preparation in increasing pain threshold parameters was equivalent to that of the rotundine tablets.

#### 3.5.4 Effects of ZXZTCs on tail flick reaction induced by photothermal stimulation in rats

Details are presented in Table 8. The pain threshold of the rotundine tablets control group was very significantly increased 30–120 min after drug administration (*p* < 0.01); the increase in pain threshold of rats in the high-dose ZXZTCs group was significant at 30 min (*p* < 0.05) and very significantly increased at 60–90 min (*p* < 0.01). Compared with the model control group, the increase in pain threshold and percentage of pain threshold in the rotundine tablets control group was highly significant during the 30–120 min period (*p* < 0.01); the pain threshold of rats in the medium-dose ZXZTCs group was significantly increased at 60 min (*p* < 0.05); the pain threshold of rats in the high-dose ZXZTCs group was significantly increased at 30 min (*p* < 0.05) and highly significantly increased at 60 min (*p* < 0.01), and the increase in the percentage of pain threshold was significant at 30 and 120 min (*p* < 0.05) and highly significant at 60 min (*p* < 0.01). Compared with the rotundine tablets control group, the percentage of pain threshold increase of rats in low-, medium-, and high-dose ZXZTCs groups was significantly lower at 30 min (*p* < 0.05) and was highly significantly lower 60–120 min (*p* < 0.01).

Our results showed that ZXZTCs could increase the pain threshold of tail flick induced by photothermal stimulation in rats and subsequently achieve the effect of analgesia. The strength of action of the high dose was the greatest, followed by medium and low doses.

#### 3.5.5 Effects of ZXZTCs on a dysmenorrhea model induced by oxytocin in mice

Details are presented in Table 8. Compared with the model control group, the latency of writhing in the ibuprofen capsules control group and low-, medium- and high-dose ZXZTCs groups was highly significantly increased (*p* < 0.01), whereas writhing in the ibuprofen capsule control group and medium and high-dose ZXZTCs groups decreased to a highly significant extent (*p* < 0.01). Compared with the ibuprofen capsule control group, the latency of writhing of mice in the low-, medium-, and high-dose ZXZTCs groups was highly significantly shortened (*p* < 0.01); the writhing of mice was highly significantly increased (*p* < 0.01) in the low- and medium-dose ZXZTCs groups and significantly increased in the high-dose ZXZTCs group (*p* < 0.05).

Our results showed that ZXZTCs can prolong the latency of writhing, reduce the writhing, and subsequently achieve an analgesic effect in mice. The strength of action was the greatest for the high dose, followed by medium and low doses.

### 3.6 Anti-inflammatory and anti-swelling effects of ZXZTCs

#### 3.6.1 Effects of ZXZTCs on ear swelling induced by xylene in mice

Details are presented in Table 9. Compared with the model control group, ear swelling of mice in the prednisone acetate tablets control group was significantly reduced (*p* < 0.05). Our results indicated no significant effects of ZXZTCs on ear swelling induced by xylene in mice, although a trend of inhibition was observed. The dose–effect relationship (from strong to weak) was in the following order: low dose, high dose, and medium dose.

**TABLE 9:** Changes of various indices of animals in anti-inflammatory experiments (*n* = 10, $\bar{x}\pm s$ ).

Group	Dose	Ear swelling model induced by xylene in mice		Foot swelling model induced by carrageenan in rats				Increased peritoneal permeability model induced by acetic acid in mice	Granuloma model induced by cotton pellets in rats
		Ear swelling (mg)	Inhibition rate of ear swelling (%)	Swelling for 1 h (mL)	Swelling for 2 h (mL)	Swelling for 4 h (mL)	Swelling for 6 h (mL)	Absorbance values (OD values)	Granuloma in rats (mg)
Model control group	—	14.26 ± 2.78	—	0.27 ± 0.12	0.39 ± 0.14	0.48 ± 0.18	0.38 ± 0.16	0.652 ± 0.110	53.94 ± 12.02
Prednisone Acetate tablets control group	Mice: 0.01 g/kg/d Rat: 0.005 g/kg/d	11.59 ± 2.51^*^	18.72%	0.17 ± 0.09*	0.15 ± 0.10**	0.16 ± 0.11**	0.18 ± 0.10**	—	40.42 ± 11.00^*^
Chlorphenamine control group	0.002 g/kg/d	—	—	—	—	—	—	0.379 ± 0.128^**^	—
Low-dose ZXZTCs group	Mice: 0.05 g/kg/d	11.44 ± 4.51	19.78%	0.17 ± 0.09	0.22 ± 0.12*	0.22 ± 0.13**	0.15 ± 0.11**	0.538 ± 0.158	43.64 ± 13.61
	Rat: 0.025 g/kg/d								
Medium-dose ZXZTCs group	Mice: 0.2625 g/kg/d	13.41 ± 3.50	5.96%	0.23 ± 0.11	0.36 ± 0.11	0.40 ± 0.21	0.34 ± 0.13	0.509 ± 0.144^**^	40.77 ± 6.68^**^
	Rat: 0.1313 g/kg/d								
High dose ZXZTCs group	Mice: 0.5250 g/kg/d	12.07 ± 2.39	15.36%	0.19 ± 0.10	0.28 ± 0.12	0.34 ± 0.07*	0.22 ± 0.10*	0.468 ± 0.207^**^	34.29 ± 13.81^**^
	Rat: 0.2625 g/kg/d								

Note: compared with the model control group, *p < 0.05,**p < 0.01.

#### 3.6.2 Effects of ZXZTCs on paw swelling induced by carrageenan in rats

Details are presented in Table 9. Compared with the model control group, paw swelling of rats in the prednisone acetate tablets control group was significantly reduced at 1 h (*p* < 0.05), and swelling at 2, 4, and 6 h was highly significantly reduced (*p* < 0.01). The degree of swelling was significantly reduced at 2 h (*p* < 0.05) and highly significantly reduced (*p* < 0.01) at 4 and 6 h in the low-dose ZXZTCs group. Paw swelling of rats in the high-dose ZXZTCs group was significantly reduced at 4 and 6 h (*p* < 0.05). Our results indicate that ZXZTCs effectively reduce the degree of paw swelling induced by carrageenan in rats and exert clear anti-inflammatory effects. The dose–effect relationship (from strong to weak) was in the following order: low dose, high dose, and medium dose.

#### 3.6.3 Effects of ZXZTCs on increase in peritoneal permeability induced by acetic acid in mice

Details are presented in Table 9. Compared with the model control group, absorbance values of the chlorphenamine control group and the medium- and high-dose ZXZTCs groups were highly significantly decreased (*p* < 0.01). Our findings indicate that ZXZTCs effectively inhibit the increase in capillary permeability induced by acetic acid in mice. The dose–effect relationship (from strong to weak) was in the following order: high dose, medium dose, and low dose.

#### 3.6.4 Effects of ZXZTCs on granuloma induced by cotton pellets in rats

Details are presented in Table 9. Compared with the model control group, granulomas in the prednisone acetate tablets control group and medium- and high-dose ZXZTCs groups were significantly reduced (*p* < 0.05). Our results showed that ZXZTCs could attenuate granulation tissue hyperplasia induced by cotton pellets in rats, specifically inducing inhibitory effects on the pathological changes of granulation hyperplasia in the late stage of inflammation. The dose–effect relationship (from strong to weak) was in the following order: high dose, medium dose, and low dose.

## 4 Discussion

The only medicinal material composition of ZXZTCs is *L. rotata*. In this study, UPLC-Q-TOF-MS technology was used to analyze 36 metabolites in ZXZTCs, all of which have been reported for *L. rotata* previously. Among the documented studies, La et al. (2015) identified 48 metabolites, Wang et al. (2018) identified 51 metabolites, Wu et al. (2016) identified 42 metabolites, and Zan et al. (2018) identified 30 metabolites in *L. rotata*. The common metabolite uncovered by different research groups and the present study was shanzhiside methyl ester. The comprehensive data indicated that shanzhiside methyl ester content is high in *L. rotata* and would not be lost when it is processed into ZXZTCs. A total of 11 metabolites of ZXZTCs were detected in the blood circulation of normal rats in our experiments. Among these, the main metabolites of iridoid glycosides in the blood were shanzhiside, shanzhiside methyl ester, 8-O-acetyl shanzhiside methyl ester, 7-deoxyloganic acid, and notohamosin B. Studies have shown that iridoid glycosides in *L. rotata* have good hemostatic (Feng, 2011) and analgesic (Yan, 2013) effects. Feng (2011) and Li et al. (2005) compared the hemostatic effects of various components via tail cutting and the examination of capillary coagulation after intragastric administration into mice. The results revealed markedly shortened thrombin times and significantly increased fibrinogen content in the presence of iridoid glycosides. Du et al. (2014) further demonstrated that iridoid glycosides could significantly shorten bleeding time. Examination of the clinical effects by Qu (2018) revealed that iridoid glycosides had strong analgesic activity and were effective constituents of analgesic prescriptions. Among them, shanzhiside methyl ester and 8-O-acetyl shanzhiside methyl ester were representative effective analgesic constituents of iridoid glycosides and their main action sites were in the spinal cord (Xue et al., 2009; Zhu, 2013). He (2011) showed that 8-O-acetyl shanzhiside methyl ester had good procoagulant activity, which could significantly increase plasma fibrin content and inhibit fibrinolytic activity in experimental mice. In addition to iridoid glycosides, *L. rotata* also contains flavonoids. The flavonoids entering into the blood circulation primarily included eugenyl-β-d-glucopyranoside, 7-methoxyapigenin, hyperoside, luteoloside, and apigenin-7-O-β-d-glucoside. The analgesic effect of flavonoids was not as significant as that of iridoid glycosides (Zhu et al., 2012; Zheng et al., 2015). However, Zhou (2009) revealed a potential dose-dependent association of the analgesic effect of *L. rotata* with total flavonoid content, indicating that the pharmacological value of flavonoids in this plant requires further research. Another previous study (Meng et al., 2009) showed that the flavonoid and iridoid glycoside metabolites of *L. rotata* exert a certain proliferative effect on bone marrow granulocytic progenitors. Additionally, phenylethanoid glycosides are among the main metabolite constituents of *L. rotata*. Among the metabolites detected in the blood, betonyoside A belongs to phenylethanoid glycosides, which have a wide range of pharmacological properties, including antibacterial, anti-inflammatory, and immune regulation activities (Jing et al., 2006). Although the specific activity of betonyoside A in *L. rotata* has not been reported, the above effects were clearly observed. Its efficacy may be attributable to combined effects with other active constituents. In summary, metabolites of ZXZTCs entering the blood may serve as potential active constituents of *L. rotata*, which have therapeutic effects on various hemorrhages, trauma (such as fracture and soft tissue injury), postoperative analgesia, congestion headache, and inflammation conditions (Zhao, 2004). In this study, an effective method for determining the contents of the six major metabolites (shanzhiside methyl ester, chlorogenic acid, 8-O acetyl shanzhiside methyl ester, forsythin B, luteoloside, and verbascoside) of ZXZTCs was established and provided a more reliable basis for the quality control and regulation of medicinal ZXZTCs. Compared with earlier literature, the average contents of the corresponding metabolites in ZXZTCs (shanzhiside methyl ester, 1.81 mg/g; chlorogenic acid, 1.43 mg/g; 8-O-acetyl shanzhiside methyl ester, 3.39 mg/g; forsythin B, 5.46 mg/g; luteoside, 2.30 mg/g; and verbascoside 1.90 mg/g) were between the lowest and highest values reported for *L. rotata* (slightly closer to the lowest reported contents). For example, Zhong et al. (2014) reported contents of 1.369–11.265 mg/g for shanzhiside methyl ester, 0.000–8.487 mg/g for chlorogenic acid, 0.000–14.898 mg/g for 8-O-acetyl shanzhiside methyl ester, 2.484–23.140 mg/g for forsythin B, 3.544–28.143 mg/g for verbascoside, and 0.000–10.757 mg/g for luteoside in *L. rotata*; Yi et al. (2016) documented a shanzhiside methyl ester content of 5.42–5.69 mg/g and Jin et al. (2021) reported a content of 0.50–0.58 mg/g in *L. rotata*. He et al. (2013) determined the contents of forsythin B, verbascoside, and luteoside in *Lamiophlomis rotate* as 0.430–6.782 mg/g, 0.661–8.600 mg/g, and 1.320–6.877 mg/g, respectively. These differences in the metabolite contents of *L. rotata* are mainly attributable to differences in origin, variety, and medicinal parts across studies. Therefore, it is more reliable to use the same *L. rotata* medicinal materials under the same preparation conditions for comparative analysis, which will provide a direction for future research on the quality of ZXZTCs. Compared with Duyiwei capsules, the corresponding metabolite contents of ZXZTCs are relatively low. Gao et al. (2015) determined the average contents of shanzhiside methyl ester, 8-O-acetyl shanzhiside methyl ester, luteoside, and verbascoside in three batches of Duyiwei soft capsules as 3.51 mg/g, 10.40 mg/g, 3.41 mg/g, and 3.60 mg/g, respectively. Overall, the quality control level of ZXZTCs needs to be improved, particularly its processing technology, on the premise of ensuring consistency of content and efficacy of the constituents.

In the present pharmacodynamic experiments, ZXZTCs have a clear hemostatic effect in different animal model states and diseases, which is manifested by shortened bleeding time, reduced platelet aggregation, and thrombosis. The mechanisms underlying the shortening of bleeding time may be related to the effects on the functions of platelets and capillaries, such as increasing the number of platelets, promoting the release of procoagulant substances from platelets, constricting local blood vessels, and decreasing capillary permeability (Lu et al., 2016). In addition, potential pharmacological activity was observed, specifically in terms of promoting the blood circulation effect. We speculate that ZXZTCs contain two main constituent types that exert procoagulant or anticoagulant activity. Shortened plasma recalcification time and prolonged thrombin time are representative of potential procoagulant and anticoagulant activities, respectively. However, the final coagulation effect of ZXZCTs was not examined in this study, and the mechanisms and metabolites that affect the coagulation system need to be further explored. We further hypothesize that thrombin time is primarily related to the coagulation, anticoagulation, and fibrinolytic system functions, and the plasma recalcification time predominantly determines the effect of drugs on the internal coagulation system. The effects of drugs on any one of the coagulation factors may influence the time of blood coagulation. In view of the theory of traditional Chinese medicine of ‘pass without pain’, the chemical constituents with anticoagulant activity may primarily be flavonoids, such as luteoloside, which exert analgesic effects by promoting blood circulation. The current study also demonstrated that the analgesic effect of ZXZTCs is mainly manifested in the response to several physical, chemical, photothermal, and other stimuli, whereas the anti-inflammatory effect is exerted primarily following exposure to acute and chronic inflammatory physical and chemical stimuli.

## 5 Conclusion

Based on previous literature on data mining, UPLC-Q-TOF-MS was used to analyze 36 metabolites in ZXZTCs, including 13 iridoid glycosides, nine flavonoids, nine phenylethanol glycosides, four phenylpropanoids, and one other metabolite. A total of 11 main metabolites of ZXZTCs were detected in the blood of normal rats, including five iridoid glycosides, five flavonoids, and one phenylethanol glycoside. Quantitative analysis of the six main metabolites (shanzhiside methyl ester, chlorogenic acid, 8-O-acetyl shanzhiside methyl ester, forsythin B, luteoloside, and verbascoside) in ZXZTCs was further conducted using HPLC. The method established in this study was simple, accurate, convenient, and reproducible, laying a foundation for improving the quality standard of ZXZTCs. Furthermore, our results on the hemostasis, analgesia, anti-inflammation, and anti-swelling effects of ZXZTCs can provide a valuable reference for its rational clinical application.

## Data Availability

The original contributions presented in the study are included in the article/Supplementary Material, further inquiries can be directed to the corresponding authors.
