# Supplementary material for: Qualitative and quantitative analyses of chemical constituents in vitro and in vivo and systematic evaluation of the pharmacological effects of Tibetan medicine Zhixue Zhentong capsules
Source: Front Pharmacol. 2023 Jul 17;14:1204947. doi: 10.3389/fphar.2023.1204947 (PMC10389267; doi:10.3389/fphar.2023.1204947)
Supplement: Supplementary file 1 [file Table1.docx]

**Supplementary table 1 Linear regression equation and correlation coefficient analysis results of 6 metabolites in ZXZTC**

| **Chemical name** | **Linear regression equation** | **Correlation coefficient (r)** | **Linear range (µg/mL)** | **Precision**  **RSD (%)** | **Repeatability**  **RSD (%)** | **Stability**  **RSD (%)** |
| --- | --- | --- | --- | --- | --- | --- |
| Shanzhiside methyl ester | y = 41.416x + 10.008 | 0.9998 | 2.675-42.8 | 0.23 | 0.7908 | 1.68 |
| Chlorogenic acid | y = 50.606x + 53.902 | 0.9992 | 4.056-64.9 | 0.67 | 0.9163 | 2.06 |
| 8-O-Acetyl shanzhiside methyl ester | y = 28.159x + 1.1157 | 0.9998 | 3.581-57.3 | 0.24 | 0.7328 | 1.32 |
| Forsythin B | y = 12.936x + 10.415 | 0.9999 | 10.8-172.8 | 0.63 | 0.6017 | 1.85 |
| Luteoloside | y = 48.958x - 12.969 | 1 | 6.937-111 | 0.25 | 0.5267 | 1.52 |
| Verbascoside | y = 28.237x + 38.085 | 0.9993 | 8.25-132 | 0.96 | 2.7353 | 1.99 |
